# Evidence for Faster X Chromosome Evolution in Spiders

**DOI:** 10.1093/molbev/msz074

**Published:** 2019-03-26

**Authors:** Jesper Bechsgaard, Mads Fristrup Schou, Bram Vanthournout, Frederik Hendrickx, Bjarne Knudsen, Virginia Settepani, Mikkel Heide Schierup, Trine Bilde

**Affiliations:** 1 Department of Bioscience, Aarhus University, Aarhus C, Denmark; 2 Department of Biology, Lund University, SE-223 62 Lund, Sweden; 3 Evolution and Optics of Nanostructure Group (EON), Biology Department, Ghent University, Ghent, Belgium; 4 Royal Belgian Institute of Natural Sciences, Brussels, Belgium; 5 Terrestrial Ecology Unit (TEREC), Biology Department, Ghent University, Ghent, Belgium; 6 Qiagen Bioinformatics, Aarhus, Denmark; 7 Bioinformatics Research Centre (BiRC), Aarhus University, Aarhus C, Denmark

**Keywords:** sex chromosome, social spider, faster-X, female bias

## Abstract

In species with chromosomal sex determination, X chromosomes are predicted to evolve faster than autosomes because of positive selection on recessive alleles or weak purifying selection. We investigated X chromosome evolution in *Stegodyphus* spiders that differ in mating system, sex ratio, and population dynamics. We assigned scaffolds to X chromosomes and autosomes using a novel method based on flow cytometry of sperm cells and reduced representation sequencing. We estimated coding substitution patterns (d*N*/d*S*) in a subsocial outcrossing species (*S. africanus*) and its social inbreeding and female-biased sister species (*S. mimosarum*), and found evidence for faster-X evolution in both species. X chromosome-to-autosome diversity (piX/piA) ratios were estimated in multiple populations. The average piX/piA estimates of *S. africanus* (0.57 [95% CI: 0.55–0.60]) was lower than the neutral expectation of 0.75, consistent with more hitchhiking events on X-linked loci and/or a lower X chromosome mutation rate, and we provide evidence in support of both. The social species *S. mimosarum* has a significantly higher piX/piA ratio (0.72 [95% CI: 0.65–0.79]) in agreement with its female-biased sex ratio. *Stegodyphus mimosarum* also have different piX/piA estimates among populations, which we interpret as evidence for recurrent founder events. Simulations show that recurrent founder events are expected to decrease the piX/piA estimates in *S. mimosarum*, thus underestimating the true effect of female-biased sex ratios. Finally, we found lower synonymous divergence on X chromosomes in both species, and the male-to-female substitution ratio to be higher than 1, indicating a higher mutation rate in males.

## Introduction

In many species with chromosomal sex determination systems, males are hemizygous for the sex chromosomes and loci harbored on sex chromosomes may therefore evolve faster than similar loci on the autosomes, an effect termed “faster-X.” Faster-X is caused by an elevated nonsynonymous substitution rate on the X chromosome if 1) new advantageous mutations are, on average, at least partially recessive, because recessive alleles are exposed to selection in the hemizygous state; and/or 2) if selection is less efficient against deleterious mutations on X chromosomes because of the smaller effective population size of the X chromosomes compared with the autosomes (Charlesworth et al. 1987; Vicoso and Charlesworth 2006; Hedrick 2007; Ellegren 2009; Wright et al. 2015). Empirical data provide conflicting conclusions on the existence and generality of “faster-X” evolution of X chromosomes (see supplementary table 1, Supplementary Material online). For example, consistent evidence of faster-X evolution comes from studies on mammals (Lu and Wu 2005; Torgerson and Singh 2006; Carneiro et al. 2012; Hvilsom et al. 2012; Xu et al. 2012) and birds (faster-Z) (Mank et al. 2007, 2010; Wright et al. 2015). Conversely, in *Drosophilids* a number of studies provide inconsistent evidence for faster-X (Betancourt et al. 2002; Counterman et al. 2004; Thornton et al. 2006; Hu et al. 2013), potentially due to low power (Charlesworth et al. 2018), and the same is true for the few other insect species studied (Jaquiery et al. 2012, 2018; Sackton et al. 2014; Rousselle et al. 2016). Different explanations proposed for this inconsistency includes both selective and ecological forces. A useful approach to study the forces causing variation in the evolution of X chromosomes is the study of closely related species that differ in traits predicted to affect X chromosome to autosome (X/A) divergence. For example, differences in life history traits and mating system, such as age at sexual maturity and polyandry, are proposed to underlie differences in X/A divergence of silent and coding sites among four primate species (Xu et al. 2012), but the number of such comparative studies are still very limited.

Populations always carry fewer X chromosomes than autosomes, and under the neutral expectation this leads to relatively fewer recombination events and higher rates of drift, which in turn decreases nucleotide diversity on X-chromosomes (Ellegren 2009; Ellegren and Galtier 2016). Based solely on the relative numbers of X chromosomes to autosomes in species with equal sex ratio, the diversity of the X chromosome is predicted to be 0.75 of that of the autosomes (Ellegren 2009). However, because the relative diversity of X chromosomes to autosomes (piX/piA) is influenced by different evolutionary forces including different mutation rates on X chromosomes and autosomes, population size fluctuations, breeding system, and recombination rate, piX/piA may deviate from 0.75 (Ellegren 2009). Disentangling the relative influence of these forces on the diversity on X chromosomes versus autosomes is important for our understanding of how molecular evolution shapes genomes (Charlesworth et al. 1987; Miyata et al. 1987; Ellegren 2007, 2009; Pool and Nielsen 2007, 2008). Deviation from the null expectation of X chromosome diversity of 0.75 of autosomal diversity is often used to infer evolutionary history. For example, piX/piA estimates of <0.75 in non-African populations of both humans and *Drosophila* were interpreted to be caused by founder events associated with “out of Africa” dispersal (Pool et al. 2012; Arbiza et al. 2014). In *Drosophila*, different sex ratios in African (unbiased) and European (male biased) populations were inferred by contrasting polymorphism data from X chromosomes and autosomes (Hutter et al. 2007).

**Fig. 1.**
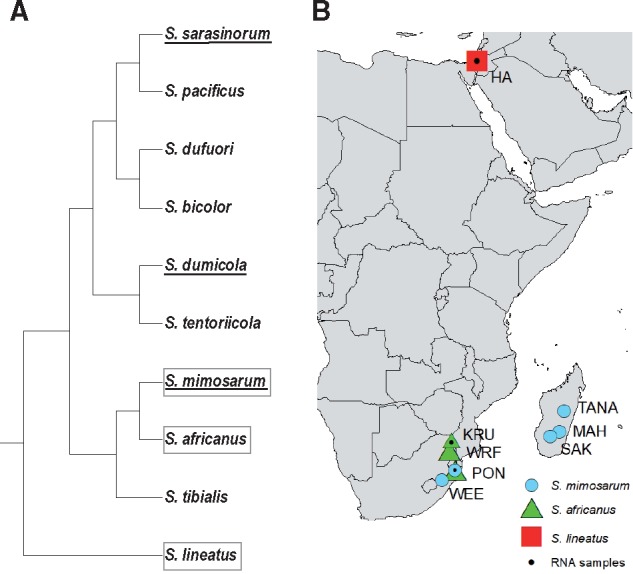
Study system. (*a*) Phylogeny of the spider genus *Stegodyphus* (modified after Settepani et al. 2016). Social species are underlined, and the species included in this study are boxed in gray. (*b*) Geographic location of sampled *S. mimosarum* populations (MAH, SAK, TANA, WEE, PON) and of *S. africanus* populations (WRF, PON, KRU).

Comparisons of closely related species have proven useful for elucidating genetic consequences of biological differences, because of their recently shared evolutionary history (Cutter et al. 2008; Guo et al. 2009; Settepani et al. 2017). Here, we present a study of two sister species with contrasting mating systems from the spider genus *Stegodyphus*: the subsocial outcrossing species *S. africanus* and its social inbreeding sister species *S. mimosarum*, with the subsocial outcrossing *S. lineatus* as an outgroup (fig. 1) (Johannesen et al. 2007; Settepani et al. 2016). The aim is to investigate how differences in biology and mating system may influence the evolution of autosomes and sex chromosomes. *Stegodyphus* spiders have an X0 sex determining system, where females have two copies of two X chromosomes (X_1_X_2_/X_1_X_2_) and males have one copy of the two X chromosomes (X_1_X_2_/0) (Forman M, personal communication). Differences in their degree of sociality and mating system, and associated life histories and population dynamics, are expected to influence substitution and diversity patterns of X chromosomes and autosomes differently: The subsocial outbreeding *S. africanus* has an equal primary sex ratio (Vanthournout et al. 2018), and populations are expected to be relatively stable in sizes and existence over evolutionary time (Lubin and Bilde 2007; Settepani et al. 2017). In contrast, the social obligatory inbreeding *S. mimosarum* shows a highly female-biased primary sex ratio (Lubin and Bilde 2007), caused by male production of a higher proportion of X_1_X_2_-containing sperm cells than sperm cells without X chromosomes (Vanthournout et al. 2018). Furthermore, empirical data suggest that population extinction rates in social *Stegodyphus* species such as *S. mimosarum* are high (Crouch and Lubin 2001; Bilde et al. 2007), implying a high rate of population colonization (Bilde et al. 2007), a pattern supported by recent population genomic analyses (Settepani et al. 2017). Differences in sex ratio influence the relative effective population sizes of X chromosomes and autosomes. If sex ratio is female biased, as in the social *S. mimosarum*, the effective population size of X chromosomes approaches that of autosomes, predicting similar evolutionary dynamics on X and A. This effect will be counter-acted if the operational sex ratio is less female biased because of female reproductive skew and cooperative breeding (Lubin and Bilde 2007; Salomon and Lubin 2007; Junghanns et al. 2017). Population size fluctuation is also an important factor, as population size reduction is predicted to more rapidly reduce X chromosome diversity relative to autosome diversity, while population growth is predicted to more rapidly elevate X chromosome diversity relative to autosome diversity. This is because population fluctuations influence effective population sizes of X chromosomes and autosomes differently, for example, Ne_X_ will experience a relatively faster decline than Ne_A_ under a population reduction (bottleneck) (Pool and Nielsen 2007).

We developed a new, cost-effective, and highly efficient approach to sort scaffolds from the *S. mimosarum* genome sequence (Sanggaard et al. 2014) into X chromosomes and autosomes using a combination of flow cytometry and reduced representation (RAD) sequencing. Subsequently, we applied transcriptome sequencing to generate estimates of X chromosome and autosome substitution patterns (d*N*/d*S*), and RAD sequencing to determine genetic diversity (pi) of X chromosomes and autosomes. With this data, we assessed theoretical predictions of how differences in sex ratio and population size dynamics affect X chromosome relative to autosome evolution in two closely related *Stegodyphus* species (*S. africanus* and *S. mimosarum*) (fig. 1).

## Results

### Assigning Scaffolds to the X Chromosome

**Fig. 2.**
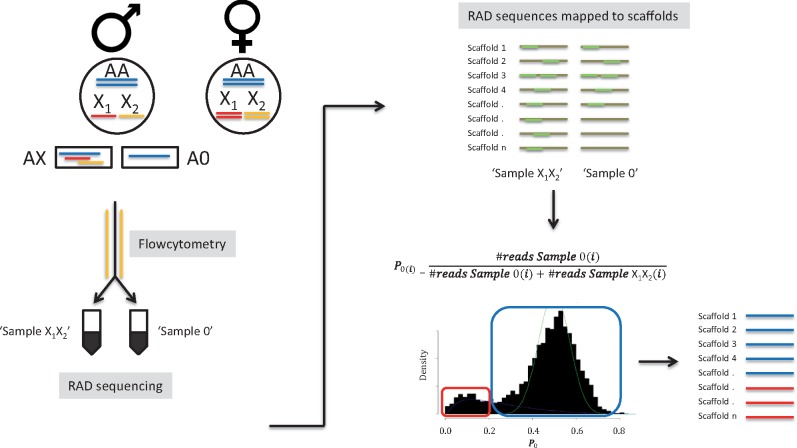
Schematic presentation of study design including assignment of scaffolds to X or autosomes. *Stegodyphus* species, like most spiders, have an X0 sex determination system, where males have only one copy of the sex chromosomes. Sperm cells were sorted into two pools using flow cytometry: one with the sex chromosomes (“Sample X_1_X_2_”) and one without the sex chromosomes (“Sample 0”), and RAD sequencing libraries from each pool were subsequently constructed and sequenced. The resulting RAD sequences from each pool were mapped to the scaffold sequences of the *S. mimosarum* genome (Sanggaard et al. 2014). Scaffolds comprising the sex chromosomes were determined as the scaffolds with no sequences (or few) mapping from the “Sample 0” pool, but with sequences mapping from the “Sample X_1_X_2_” pool. For each scaffold, we estimated a summary statistic (*P*_0_) defined as the number of reads that mapped from “Sample 0” divided by the sum of reads that mapped from both “Sample 0” and “Sample X_1_X_2_” after normalization of the total number of reads from both samples. Scaffolds belonging to X chromosomes are predicted to have *P*_0_ close to 0, while those belonging to autosomes are predicted to have *P*_0_ close to 0.5.

We were able to isolate the nuclei from sperm cells extracted from an *S. mimosarum* male pedipalp, and separate the nuclei with and without X chromosomes using flow cytometry. Using RAD sequencing of the nuclei, we obtained more than 1 million reads after quality filtering from each sample that were subsequently mapped to the reference genome of *S. mimosarum*. Figure 2 shows a density distribution of the number of reads from the sample without X chromosomes (“Sample 0”) divided by the total number of reads from both samples (“Sample 0 + Sample X_1_X_2_”) mapped to each scaffold (see Materials and Methods for details). The distribution is bimodal (fig. 2 and supplementary fig. 1, Supplementary Material online). The major peak close to *P*_0_ = 0.5 shows that most scaffolds have a similar coverage in “Sample 0” and “Sample X_1_X_2_,” while the minor peak around *P*_0_ = 0.119 constitutes scaffolds with much lower coverage in “Sample 0” compared with “Sample X_1_X_2_,” suggesting that they are placed on the X chromosomes. We used a threshold of *P*_0_ < 0.238 to select scaffolds that we assign to the X chromosomes and *P*_0_ > 0.3 as a threshold for a scaffold to be considered autosomal, with both thresholds corresponding to a FDR of 2.5% (supplementary fig. 1 and table 2, Supplementary Material online). In this way, we obtained 450 X chromosome scaffolds for downstream analyses (a list assigning scaffolds to X chromosome and autosome scaffolds can be found in supplementary table 3, Supplementary Material online).

Characterizing the X chromosome scaffolds using the *S. mimosarum* reference genome, we found 2,132 X-linked genes across 246.7 Mb or one gene per 115,725 bp (8.64 genes per Mb) compared with an average autosomal gene density of one gene per 103,622 bp (9.65 genes per Mb). The average gene length of the reference genome is 32,170 bp, while genes on X chromosome scaffolds are on average 42,638 bp long (see supplementary table 4, Supplementary Material online, for further summary statistics). We used flow cytometry data from Vanthournout et al. (2018) to estimate the proportion of the genome made up by the X chromosomes. In *S. africanus*, we estimate that the X chromosomes make up 15.3% (SD: 0.009) of the total genome, and in *S. mimosarum* it is 15.1% (SD: 0.012). We note that the identified X chromosome scaffolds make up ∼9% of the genome whereas flow cytometry indicates the X chromosomes make up 15% of the genome. Approximately half of the remaining 6% were not assigned due to too low coverage from the RAD sequencing of the nuclei, while the other half are likely to have a P_0_ above the threshold of 0.238 (supplementary fig. 1, Supplementary Material online). We found no differences in codon usage bias (Supek and Vlahovicek 2004) among genes located on X chromosomes and autosomes in any of the two species (supplementary fig. 2, Supplementary Material online).

### Substitution Patterns of Autosomes

**Fig. 3.**
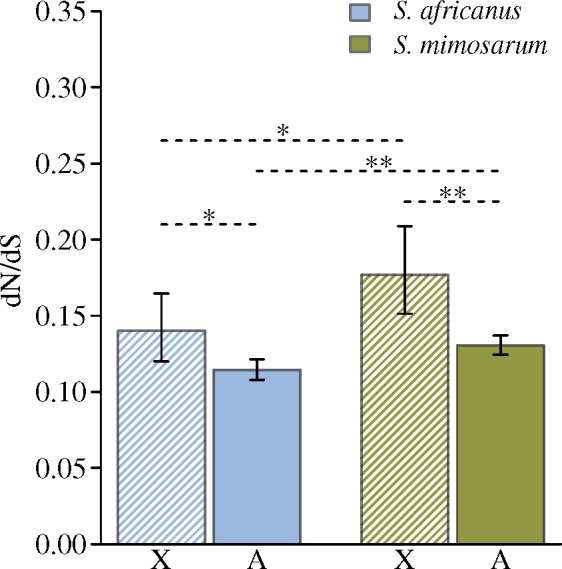
d*N*/d*S* estimates for X chromosomes and autosomes separately from *Stegodyphus africanus* and *S. mimosarum* based on consensus sequences of transcriptome data. Error bars represent 95% confidence limits obtained by bootstrapping. *P* values were estimated by randomization tests. *<0.05, **<0.01.

After processing the transcriptome sequence data, we obtained consensus sequences of 4,641 putative orthologous loci (including 523 on the X chromosomes) from all three *Stegodyphus* species, and aligned these for comparative studies. We used PAML ver. 4.6 (Yang 2007) to estimate species-specific d*N*/d*S* ratios of *S. mimosarum* and *S. africanus* using *S. lineatus* as outgroup for X chromosomes and autosomes separately. The autosomal d*N*/d*S* ratio of the social *S. mimosarum* was significantly larger than for *S. africanus* (0.131 vs. 0.114; randomization test: *P *=* *0.004; fig. 3 and table 1), suggesting stronger purifying selection in the outcrossing *S. africanus* compared with the inbreeding *S. mimosarum*. This is consistent with the estimate of a 10-fold higher effective population size in the outcrossing *S. africanus* than the inbreeding *S. mimosarum* (Settepani et al. 2017), and stronger effect of selection in populations with larger effective size to remove slightly deleterious mutations (Charlesworth 2009). A list of genes assigned to X chromosome and autosome scaffolds can be found in supplementary table 5, Supplementary Material online.

**Table 1.** d*N* and d*S* Estimates for Loci Located on the X Chromosomes and Autosome Scaffolds for *Stegodyphus africanus* and *S. mimosarum*.

	d*N* (CI_95low_−CI_95high_)	d*S* (CI_95low_−CI_95high_)	d*N*/d*S* (CI_95low_−CI_95high_)
*S. mimosarum*			
Autosomes	0.0012 (0.0012–0.0013)	0.0093 (0.0090–0.0096)	0.131 (0.125–0.137)
X chromosomes	0.0010 (0.0009–0.0012)	0.0059 (0.0052–0.0065)	0.177 (0.152–0.208)
*S. africanus*			
Autosomes	0.0010 (0.0009–0.0010)	0.0083 (0.0080–0.0086)	0.114 (0.108–0.121)
X chromosomes	0.0008 (0.0007–0.0010)	0.0060 (0.0054–0.0066)	0.140 (0.120–0.164)

Note.—*Stegodyphus lineatus* was used as outgroup. In parenthesis are 95% confidence limits that are obtained by bootstrapping.

### Substitution Patterns of X Chromosome versus Autosome

The X-linked d*N*/d*S* ratios were 0.140 in *S. africanus* and 0.177 in *S. mimosarum*, and significantly larger than the autosomal d*N*/d*S* ratios of 0.114 in *S. africanus* and 0.131 in *S. mimosarum* (randomization tests: *P *=* *0.018 and *P *=* *0.004, fig. 3 and table 1). In both species, we found a significantly lower synonymous substitution rate on the X-linked genes (d*S*_X_) compared with autosomal genes (d*S*_A_) (for *S. africanus* d*S*_X_/d*S*_A_ = 0.72; randomization test: *P *<* *0.001; for *S. mimosarum* d*S*_X_/d*S*_A_ = 0.63; randomization test: *P *<* *0.001, table 1).

### Genetic Diversity on X Chromosomes and Autosomes

**Fig. 4.**
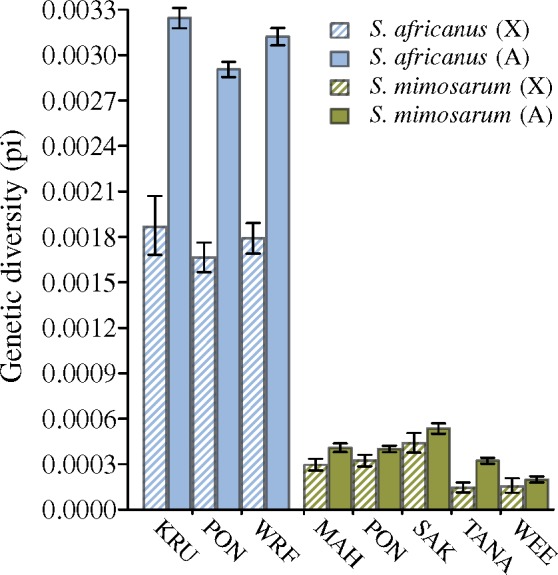
Genetic diversity estimated from RAD data for X chromosomes (X) and autosomes (A) from three populations of *Stegodyphus africanus* (WRF, PON and KRU) and five populations of *S. mimosarum* (MAH, SAK, TANA, WEE and PON). We used data from between 5 and 10 individuals per loci from each population depending on coverage. Error bars represent 95% confidence limits obtained by bootstrapping.

**Fig. 5.**
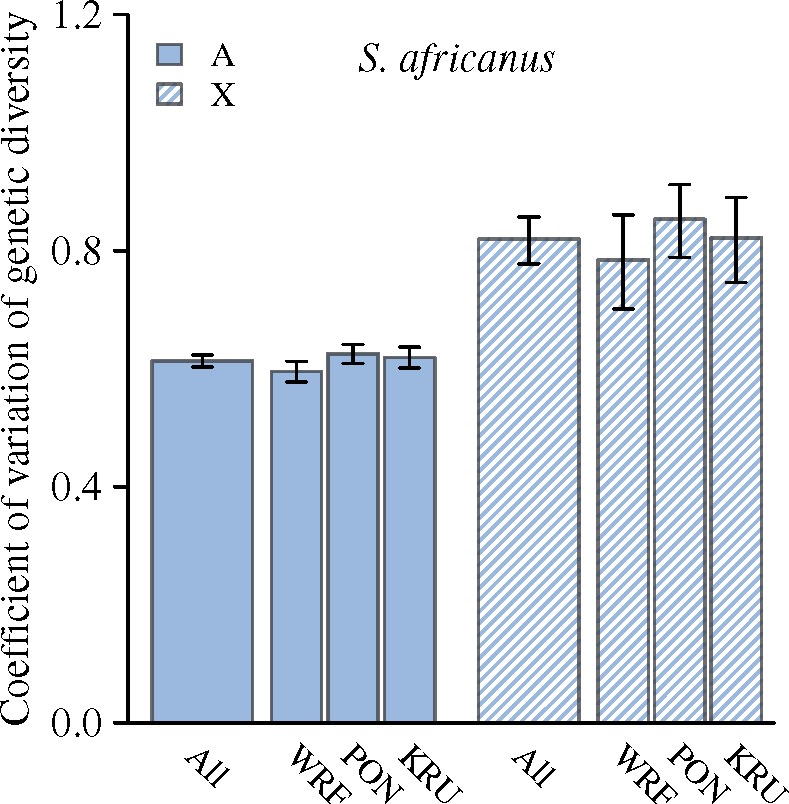
Comparison of the coefficient of variation (CV) of genetic diversity among scaffolds assigned to the X chromosomes and autosomes. Only in *Stegodyphus africanus*, was genetic diversity sufficiently large to allow this comparison. WRF, PON and KRU represent the three sampled *S. africanus* populations, while ALL is the average per species. CVs were estimated as SD/average. Error bars represent 95% confidence limits obtained by bootstrapping.

**Fig. 6.**
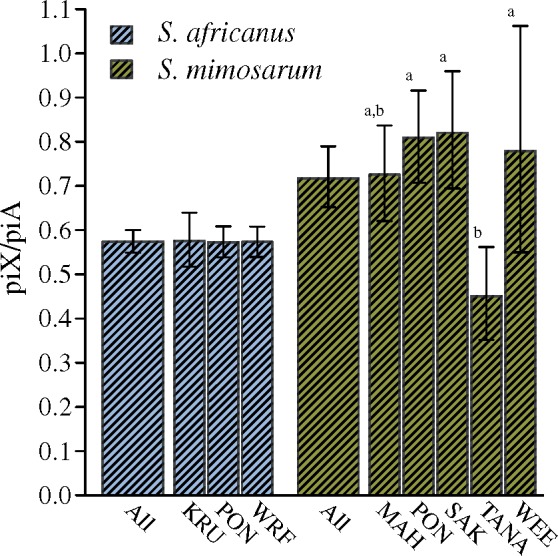
Ratios of X chromosome to autosome genetic diversity (piX/piA) based on pi estimates presented in figure 5. Estimates are presented for three populations of *Stegodyphus africanus* (WRF, PON and KRU) and five populations of *S. mimosarum* (MAH, SAK, TANA, WEE and PON), as well as the species average (ALL). Significant differences (indicated by different letters) between populations within each species were investigated using *F*-tests and Tukey’s HSD method for post hoc comparisons. Error bars represent 95% confidence limits obtained by bootstrapping.

From RAD sequencing, we obtained 24,321 RAD loci (3,440 X-linked) from the outcrossing *S. africanus* and 20,665 RAD loci (2,783 X-linked) from the inbreeding *S. mimosarum*. Using the *S. mimosarum* reference genome (Sanggaard et al. 2014) we found that 1.16% of the RAD loci are located in protein coding regions. We estimated total diversity in three *S. africanus* and five *S. mimosarum* populations, and found that all *S. mimosarum* populations have reduced diversity on both X chromosomes and autosomes compared with *S. africanus* (both reduced by ∼85%) (fig. 4). We note that the diversity estimates presented here are highly similar to those obtained by Settepani et al. (2017) who analyzed the same RAD sequence data using a different pipeline. Variation in diversity across scaffolds may reflect different rates of loss of diversity, which is in accordance with linked selection playing a predominant role in loss of diversity. In *S. mimosarum*, on average 56% of the autosome scaffolds and 61% of the X-linked scaffolds had a diversity of 0 (supplementary fig. 3, Supplementary Material online), preventing us from meaningful inference of the variation in loss of diversity across scaffolds, as the variation among these 0-diversity scaffolds is lost due to the zero boundary. This was not the case for *S. africanus*, where only 3% and 9% of autosome and X-linked scaffolds, respectively, had diversity estimates of 0 (supplementary fig. 3, Supplementary Material online), and we therefore contrasted variation in diversity across X chromosome scaffolds to autosome scaffolds by the coefficient of variation (CV). We find that diversity varies significantly more among X-linked scaffolds than among autosome scaffolds consistent with a stronger role for natural selection in removing diversity in regions of the X chromosome than on the autosomes, by either selective sweeps or background selection (fig. 5). Estimates of piX/piA are more or less constant among subsocial *S. africanus* populations, but varies significantly among social *S. mimosarum* populations (*S. africanus*: *F*_2,__99_ = 0.003; *P *=* *0.99, *S. mimosarum*: *F*_4,__146_ = 4.63; *P *<* *0.01) (fig. 6). Averaged across populations, the X to autosome diversity ratio (piX/piA) is 0.57 (95% CI: 0.55–0.60) for *S. africanus*, which is lower than the 0.75 expected with an equal contribution of the two sexes. piX/piA of *S. mimosarum*, 0.72 (95% CI: 0.65–0.79), was not significantly different from the 0.75 expected, but significantly higher than in *S. africanus* (χ^2^_(1)_ = 4.25; *P *=* *0.04) (fig. 6).

Population size fluctuations reduces effective population size on X chromosomes relatively more than on autosomes (Pool and Nielsen 2007, 2008; Schou et al. 2017). Populations of social spiders such as *S. mimosarum* undergo recurrent population size fluctuations due to propagule dispersal of single-mated females and high-population turnover rates (Bilde et al. 2007; Settepani et al. 2014, 2017). Population size fluctuations can therefore potentially explain the fluctuating piX/piA among *S. mimosarum* populations. We used simulations to quantitatively investigate the effects of recurrent population size fluctuations on piX/piA using fastsimcoal2 (Excoffier et al. 2013). We found that recurrent population size fluctuations with realistic parameters for *S. mimosarum* can significantly reduce piX/piA (supplementary fig. 4, Supplementary Material online). piX/piA is influenced more by few founders (5 chromosomes vs. 50 chromosomes) and to a lesser extent by slower growth after a founder event (100 generations vs. 50 generations between founder events).

A McDonald–Kreitman test was performed using polymorphisms from the *S. mimosarum* RAD loci located in protein coding genes and divergences from the transcriptome sequences. In total, we identified 150 synonymous and 140 nonsynonymous polymorphisms at the X chromosomes and 1,433 synonymous and 1,198 nonsynonymous polymorphisms at the autosomes. In the *S. mimosarum* lineage, we identified 563 synonymous and 337 nonsynonymous substitutions at the X chromosomes and 15,599 synonymous and 7,370 nonsynonymous substitutions at the autosomes. Alpha estimates were estimated to be negative for both X chromosomes and autosomes (−0.56 and −0.77, respectively), suggesting that many nonsynonymous mutations are segregating likely because they are slightly deleterious.

### X Chromosome Substitution Rates and Sex-Biased Mutation Rates

We find that the synonymous divergence of X chromosomes is lower than for autosomes in both species estimated from transcriptome data (d*S*_X_/d*S*_A_ 0.72 in *S. africanus* and 0.63 in *S. mimosarum*; table 1). This divergence ratio does not account for differences in coalescence times of X chromosomes and autosomes caused by differences in effective population size in the ancestor of *S. africanus* and *S. mimosarum*. Since the effective population size of X chromosomes is smaller than that of autosomes, the X chromosomes are expected to coalesce faster than autosomes in the ancestral species. The difference in synonymous divergence estimates of X chromosomes and autosomes is therefore not solely due to different mutation rates, but also different times to accumulate substitutions. To correct for different coalescence times in the ancestral species, we assumed an ancestral population size (*N*_A_) of 300,000 (Settepani et al. 2017), a sex ratio of 1:1, and species split time of 1 My (using the mutation rate from Mattila et al. 2012). Under these assumptions, the predicted time to coalescence of X chromosomes is 85% of that of the autosomes (see supplementary fig. 5, Supplementary Material online). Based on the adjusted d*S*_X_/d*S*_A_ divergence ratio from transcriptome data (*S. africanus*: 0.85, *S. mimosarum*: 0.74), we estimate the male-to-female substitution ratio (α) (Miyata et al. 1987) to 2.6 in *S. africanus* and 8.1 in *S. mimosarum*. In addition, we calculated a synonymous divergence ratio based on RAD data (dRAD_X_/dRAD_A_) in *S. mimosarum*, taking advantage of the fact that the Madagascan and South African populations are genetically isolated from each other. The estimated dRAD_X_/dRAD_A_ divergence ratio is 0.85, and 0.89 when adjusting for different coalescence times in the ancestral population (supplementary fig. 5, Supplementary Material online). Using the adjusted dRAD_X_/dRAD_A_ divergence ratio, we get an α estimate of 1.98.

## Discussion

The method used to identify X-linked scaffolds in this study is applicable for species with X0 or heterogametic sex determination, where X chromosomes are sufficiently large for sperm cells with and without the X chromosomes to be separated using flow cytometry. Large full-genome sequencing initiatives to sequence 5,000 insect and insect-related genomes (i5K) (Evans et al. 2013), and the Global Invertebrate Genomics Alliance (GIGA) (Bracken-Grissom et al. 2014) can directly benefit from our approach and allow a large number of sex chromosome systems to be investigated in order to disentangle hypotheses regarding their involvement in meiotic drive (Jaenike 2001; Unckless et al. 2015), sexual conflict (Andres and Morrow 2003; Mank et al. 2014), and speciation (Presgraves 2008; Kitano et al. 2009).

### Faster-X Evolution in *S. mimosarum* and *S. africanus*

We found evidence for faster-X evolution in both *S. mimosarum* and *S. africanus*, providing the first case of faster-X evolution in spiders (see supplementary table 1, Supplementary Material online, for a survey of previous faster-X investigations) (Garrigan et al. 2014; Kousathanas et al. 2014; Sackton et al. 2014). Faster-X can be caused by drift or adaptive substitutions at the X chromosomes. To test if faster-X is caused by adaptive evolution, we used transcriptome data and RAD sequences located in exons to estimate the proportion of substitutions that are fixed by adaptive evolution using the McDonald–Kreitman test (McDonald and Kreitman 1991). Negative α values were obtained for both X chromosomes and autosomes, suggesting that slightly deleterious mutations segregate. We can therefore not conclude from this analysis to which extent faster-X is caused by drift or adaptive evolution. Estimating the proportion of adaptive substitution in the presence of segregating slightly deleterious mutations would require targeted sequencing of protein coding loci in multiple individuals (Eyre-Walker and Keightley 2009). Two other observations from our data are however informative and consistent with adaptive evolution contributing to faster-X in this system. The effective population size of *S. mimosarum* was reduced by ∼90% during the evolution of social behavior (Settepani et al. 2017). Such an increase in genetic drift has caused an increase in autosomal d*N*/d*S* of only 15% (0.131 vs. 0.114). In comparison, a much lower difference in effective population size of X chromosomes and autosomes is associated with substantial increase in d*N*/d*S* of 35% (0.177 vs. 0.131) in *S. mimosarum* and 22% (0.140 vs. 0.114) in *S. africanus*, supporting that the increase in d*N*/d*S* of X chromosomes is not only caused by genetic drift. Adaptive evolution is further supported by the finding that diversity along the X chromosomes varies more than along the autosomes in *S. africanus*, suggesting that selective sweeps are more prominent on the X chromosomes, a phenomenon also observed in primates (Nam et al. 2017). Finally, in support of a prominent role of drift causing faster-X, we find Ne_X_/Ne_A_ < 0.75 in *S. africanus* (as estimated by piX/piA). However, if the difference in diversity on X (piX) and A (piA) is caused by a lower mutation rate at the X chromosomes and not drift, this is unlikely to have an effect on adaptive substitutions (Vicoso and Charlesworth 2009). Indeed our data suggests a lower X chromosome mutation rate (see X Chromosome Mutation Rate below), and the effects of genetic drift on d*N*/d*S* may not be as strong as suggested by the deviation of Ne_X_/Ne_A_ from 0.75.

An alternative and nonexclusive explanation of faster-X is a lower recombination rate of X chromosomes compared with autosomes, arising as X chromosomes unlike autosomes only recombine in females. A reduced recombination rate on X chromosomes is predicted to increase the effect of linked selection, which would increase d*N*/d*S* due to fixation of slightly deleterious mutations. This should produce a negative correlation between recombination rate and rate of nonsynonymous substitutions, as reported in, for example, *Drosophila* (Assis et al. 2012).

The potential for “faster-X” evolution depends on the difference between the effective population sizes of X chromosomes (Ne_X_) and autosomes (Ne_A_). In species with a female-biased sex ratio as observed in *S. mimosarum*, the difference between Ne_X_ and Ne_A_ is expected to be lower compared with species with equal sex ratio. Such a scenario provides a wider range of dominance levels where beneficial mutations at the X chromosomes are more rapidly fixed (Vicoso and Charlesworth 2009), making species with biased sex ratio more prone to “faster-X” evolution. However, according to our diversity estimates of X chromosomes and autosomes, we do not find support for intensified faster-X in *S. mimosarum* relative to *S. africanus* (*S. mimosarum*, X_(d__*N*__/d__*S*__)_/A_(d__*N*__/d__*S*__)_: 1.35, *S. africanus*, X_(d__*N*__/d__*S*__)_/A_(d__*N*__/d__*S*__)_: 1.22; *P *=* *0.92). As *S. mimosarum* was used for assignment of scaffolds to X chromosomes or autosomes, usage of the same assignment in *S. africanus* and thereby the species comparisons made above, relies on no independent rearrangements occurring between X chromosomes and autosomes. Cytogenetic analyses of *S. mimosarum* and *S. africanus* have shown that the X chromosomes appear highly similar (Forman M, personal communication), supporting the assumption that no major X chromosome rearrangements occurred since the species split.

### Genetic Diversity on X Chromosomes and Autosomes

Our previous studies showed that the social species has a much smaller effective population size and a high rate of population turnover (Settepani et al. 2014, 2017). In agreement with this, we observed considerably higher genetic diversity in *S. africanus* along with a lower d*N*/d*S* ratio suggesting that purifying selection is more efficient in the outbreeding species.

The finding of an X to autosome diversity ratio (piX/piA) in S. africanus lower than the expectation of 0.75 (no sex ratio bias) suggests that additional evolutionary forces, such as differences in mutation rates and/or selection may reduce diversity on X chromosomes at a higher rate than on autosomes. Mutation rate on the X chromosomes was inferred to be lower than on the autosomes, which at least partly explains the low piX/piA in *S. africanus*. Selection is known to cause loss of genetic diversity not only in the selected loci but also in flanking regions due to genetic hitchhiking (Smith and Haigh 1974; Begun and Aquadro 1992) and background selection (Charlesworth 2012). The effect of removing diversity by linked selection is predicted to be larger in genomic regions where recombination rates are small, as for X chromosomes that do not recombine in males. The finding of lower diversity on X chromosomes may therefore partly be due to selection. Exposure of recessive variants on X chromosomes to selection in males may enforce this effect, however, the lower effective population size of the X chromosomes may cause selection to be less efficient on X chromosome loci, potentially reducing this effect. The social *S. mimosarum* has a primary female-biased sex ratio (Lubin and Bilde 2007; Vanthournout et al. 2018), so a higher piX/piA is expected compared with the subsocial *S. africanus* if the operational sex ratio is also female biased (Ellegren 2009). In agreement with this expectation, piX/piA in *S. mimosarum* was higher than in *S. africanus*. We propose that this is due to similar evolutionary forces as discussed for *S. africanus*, which decrease piX/piA, and the additional effect of female bias that increases piX/piA.

**Fig. 7.**
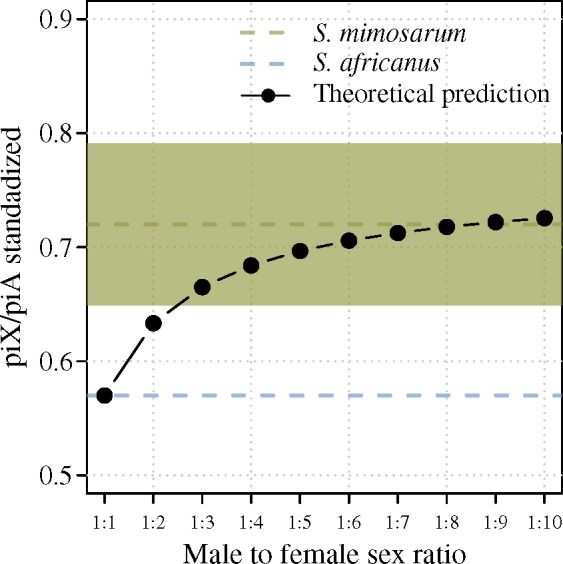
Theoretically expected piX/piA as a function of male to female sex ratio (black dots). The expected piX/piA is standardized according to the finding that *Stegodyphus africanus* has a lower piX/piA than the expected 0.75, which we infer to be caused by a lower X chromosome mutation rate and the effect of linked selection, and assume to have a similar effect in *S. mimosarum*. Point estimates of piX/piA for the two species are depicted with dotted lines, and for *S. mimosarum*, the 95% confidence limits of this estimate are shown.

Social spiders are cooperative breeders with reproductive skew so only a fraction of females reproduce (Lubin and Bilde 2007; Junghanns et al. 2017), but it is currently unclear how large a proportion of females that reproduce, and therefore what the operational sex ratio is. With everything else equal, using the difference in piX/piA between the two species makes it possible to estimate the operational sex ratio. The point estimate of piX/piA (0.72) is consistent with an operational female bias between 1:8 and 1:9, and the lower boundary of the confidence limits suggests that the operational female bias is stronger than 1:2 (fig. 7). However, previous studies suggest that the population sizes of social species fluctuate substantially due to recurrent founder events associated with population extinction/recolonization dynamics (Crouch and Lubin 2001; Bilde et al. 2007; Settepani et al. 2017). Population size fluctuations affect diversity on X chromosomes more than diversity on autosomes, and therefore also the piX/piA ratio due to the Pool–Nielsen effect (Pool and Nielsen 2007). We simulated recurrent founder events and showed that piX/piA values constantly lower than equilibrium (estimated by simulating a constant population size) can be reached when founder events are frequent. Depending on the stage in the Pool–Nielsen cycle following a founder event at which the dynamic equilibrium is modeled, population dynamics with recurrent founder events would explain the variation observed in piX/piA among the *S. mimosarum* populations. The actual effect of female bias on piX/piA may therefore be larger than we observed due to a possible counteracting effect of population size fluctuations, and consequently the operational sex ratio even more female biased (supplementary fig. 6, Supplementary Material online).

### X Chromosome Mutation Rate

We found lower synonymous divergence on X chromosomes compared with autosomes suggesting a lower mutation rate on X than for the autosomes in both species. Importantly, this finding persisted when taking differences in coalescence time of X chromosomes and autosomes in the ancestral species into account. A lower mutation rate on X chromosomes can have several causes. One possibility is that the mutation rate is lower on the X chromosomes simply due to different sequence composition of the X chromosomes and autosomes. However, to our knowledge there is no evidence from previous studies that this is a plausible explanation. Another possible cause is a lower recombination rate of X chromosomes, which only recombine in females, than of autosomes which recombine in both sexes. Since recombination can be a source of mutations (Arbeithuber et al. 2015), the mutation rate on X chromosomes is expected to be lower than on autosomes, but the overall effect is not expected to be very high due to the relatively low number of recombination events on the X-chromosome per generation. Another possibility is that mutations are male-biased. Since X chromosomes spend 1/3 of their time in males and 2/3 in females, a male-biased mutation rate will cause a lower mutation rate at X chromosome compared with autosomes. A male-biased mutation rate has been found in several vertebrate species, and has been interpreted to be due to more cell divisions in spermatogenesis than in oogenesis. However, in short lived species like spiders and other invertebrates, the number of cell divisions in spermatogenesis and oogenesis is often similar, like in, for example, *Drosophila* (Drost and Lee 1998). Recent evidence mainly from humans suggests an alternative cause of a male-biased mutation rate, namely that mutation rate simply is higher in spermatogenesis than in oogenesis. No evidence for such an effect in spiders exists, but as we find none of the aforementioned explanations to be convincing, we suggest this alternative as a possibility.

## Conclusions

This first analysis of DNA sequence evolution of X chromosomes in spiders reveals faster-X evolution in two sister-species that differ in mating systems, population dynamics and sex ratio bias. The extent of faster-X evolution is similar in the two species, contrary to theoretical predictions when sex ratios diverge from 50% to 50%. Contrasting the relative genetic diversity on X chromosomes and autosomes in a social inbreeding and a subsocial outcrossing species revealed higher piX/piA, and larger variation of piX/piA among populations in the social inbreeding species. These findings are consistent with the effects of female bias and Pool–Nielsen effects caused by frequent population size fluctuations in the social inbreeding *S. mimosarum*. Finally, we infer that the X chromosome mutation rate is lower than the autosome mutation rate in both species, potentially caused by a higher mutation rate in spermatogenesis than in oogenesis.

## Materials and Methods

### Study System

The spider genus *Stegodyphus* (family Eresidae) contains more than 20 species. Three of the species have an independently derived social behavior (fig. 1) (Johannesen et al. 2007; Settepani et al. 2016), which is consistently associated with a female-biased sex ratio, reproductive skew and an inbreeding mating system, also named the “social syndrome” (Lubin and Bilde 2007). Family groups of social species live and breed in closed nests that propagate within populations by nest fission and by long distance dispersal through ballooning of mated females (Lubin and Bilde 2007). In comparison, the subsocial species have equal sex ratios, no reproductive skew and are outcrossing (Bilde et al. 2005; Lubin and Bilde 2007).

### Data Sets

#### RAD Sequence Data of Sperm Cells

To allocate reference scaffolds to an autosome or an X chromosome, we used flow cytometry (Garner et al. 2013) to sort free nuclei from *S. mimosarum* sperm cells, and subsequently RAD sequencing the DNA (fig. 2). Free nuclei from sperm cells were obtained by trypsin treatment and their DNA was stained with propidium iodide (Vindelov et al. 1983; Aron et al. 2003; Vanthournout et al. 2014). The nuclei were sorted based on DNA content into on a BD Biosciences FACSAria cell sorter (Argon laser emitting at 488 nm), into a sample with the two X chromosomes (“Sample X_1_X_2_”) and one without the two X chromosomes (“Sample 0”). From each of the two samples, paired-end RAD sequencing libraries were constructed using the protocol described in (Poland et al. 2012), with the following modifications: 0.5 µl of BSA was added to the Restriction Mastermix and an AMPure Beads clean-up and size selection step was implemented after PCR amplification. The libraries were sequenced using the Illumina HiSeq 2000 platform (100 bp paired-end).

#### Transcriptome Sequence Data

To enable inference of the coding substitution patterns between *S. africanus* and *S. mimosarum*, we obtained transcriptomes of the two species as well as an outgroup species (*S. lineatus*). Libraries of an *S. lineatus* and an *S. africanus* female were constructed using Illumina’s TruSeq Stranded mRNA LT Sample Prep Kit, and sequenced on an Illumina HiSeq2000 platform (100 bp paired-end). For *S. mimosarum*, quality filtered transcriptome data, also sequenced on an Illumina HiSeq 2000 platform (100 bp paired-end), from a previous study was used (Sanggaard et al. 2014).

#### RAD Sequence Data of Populations

To estimate molecular diversity of autosomes and X chromosomes in *S. mimosarum* and *S. africanus*, we used quality filtered RAD sequenced reads (100 bp paired-end) from a previously published study (Settepani et al. 2017). This data set contained individual data from 49 *S. mimosarum* females (each sampled from its own distinct nest) and 27 *S. africanus* females, with an average of 3.6 million clean reads per individual. The *S. mimosarum* females were sampled from five populations (fig. 1); ten from each of four populations (MAH, SAK, TANA, WEE) and nine from one population (PON). Three of the *S. mimosarum* populations are located in Madagascar (MAH, SAK, TANA) and two in South Africa (WEE, PON). The *S. africanus* females were sampled from three South African populations (WRF, PON, KRU) (fig. 1), with eight, ten, and nine, respectively).

### Data Analyses

#### Identifying Scaffolds from the X Chromosomes

The RAD sequence reads from the sorted sperm cells were quality trimmed using the FASTX toolkit (http://hannonlab.cshl.edu/fastx_toolkit). We discarded reads containing a base with a Phred quality score <10, as well as reads with an average Phred quality score <30. More clean reads were obtained from “Sample 0” than “Sample X_1_X_2_,” and it was therefore subsampled to obtain same number of reads from both samples (1,034,261 reads). The clean data from the two samples (“Sample 0” and “Sample X_1_X_2_”) were mapped separately to the reference genome sequence of *S. mimosarum* (Sanggaard et al. 2014), using CLC Genomics Workbench 7 (default parameters). The reference genome consists of ∼23,000 scaffolds (N50 = 480,636 bp) and 45,000 contigs (N50 = 17,272 bp). Scaffolds for which at least 100 reads from “Sample X_1_X_2_” mapped (3,490 in total), was considered to have a potential X chromosome origin. The number of reads mapped from “Sample 0” was hereafter divided by the sum of the corrected number of reads mapped from “Sample 0” and “Sample X_1_X_2_.” We call this proportion *P*_0_.

$$P_{0}(i)=\frac{\#\mathrm{reads}\mathrm{Sample}0(i)}{\#\mathrm{reads}\mathrm{Sample}0(i)+\#\mathrm{reads}\mathrm{Sample}X_{1}X_{2}(i)}.$$

Given a high-quality sorting of nuclei, we expect a bimodal distribution of *P*_0_. One mode will contain the distribution for the scaffolds belonging to the X chromosomes and the other will contain the distribution for the scaffolds belonging to the autosomes. The first distribution is expected to have an average just >0, as the sorting of the two nuclei types is imperfect. The second distribution is expected to have an average just >0.5, since the reads from “Sample X_1_X_2_” will be mapped to more scaffolds (both X chromosomes and autosomes scaffolds) than the reads from “Sample 0” (only autosomes). How much >0.5 depends on the distribution of RAD loci on X chromosomes and autosomes, and the precision of the sorting of the two nuclei types. A Bayesian mixture analysis was performed in order to separate the two distributions and estimate their proportion, mean, and variance (fig. 2; see also Supplementary Material online). The two identified distributions overlapped slightly, with 0.119 and 0.500 as the means of the distribution predicted to be composed of X chromosome and autosome scaffolds, respectively. When determining what minimum *P*_0_ to use as a cut-off for a scaffold to be considered an autosome, we tested three different cut-offs (0.119, 0.3, and 0.5). This was necessary due to the overlap of distributions in the autosomal peak (supplementary fig. 1, Supplementary Material online). Using transcriptomic data and RAD sequencing data on each of the three cut-offs, we estimated pi and d*N*/d*S* ratio for the autosomes, respectively. These two measures were consistent across the three cut-offs, and in particular between 0.3 and 0.5 (supplementary figs. 7 and 8, Supplementary Material online). Based on this comparison, and an estimated false positive rate of 2.5% (supplementary table 2, Supplementary Material online) we continued with *P*_0_ = 0.3 as the lower cut-off for the full set of analyses. A false positive rate of 2.5% was also used as to determine the upper cut-off for assigning scaffolds as belonging to the X chromosomes (*P*_0_ = 0.239).

#### Molecular Evolution at X Chromosomes and Autosomes

The raw sequences of all three species were quality trimmed using the FASTX toolkit (http://hannonlab.cshl.edu/fastx_toolkit). We discarded reads containing a position with a Phred quality score <10, as well as reads with an average Phred quality score <30. The clean data from all three species were mapped separately to a gene list of the *S. mimosarum* genome (Sanggaard et al. 2014) consisting of 26,314 loci all beginning with a start codon (ATG) using CLC Genomics Workbench 7 (default parameters). For each species, loci with average coverage <3 were initially removed. Consensus bases were called in all positions with coverage 8 or higher, while positions with coverage between 3 and 7 were masked and not included in downstream analyses. Ambiguous bases (IUPAC) were called when a base was supported by at least three reads and/or if its proportion was >10%. Only consensus sequences with <2.5% ambiguous bases were retained. The resulting consensus sequences were grouped based on their mapping to the *S. mimosarum* gene list by assuming orthology and subsequently aligned across species using PRANK (Loytynoja and Goldman 2008). Alignments were manually edited assuming that frame shifts were caused by sequencing or assembly errors. In total, 8,302 alignments with sequences from all three species were obtained, of which 285 belonged to X chromosome scaffolds and 8,017 to autosome scaffolds. All codons that could not be translated into an amino acid for a given species (because of Ns or ambiguous nucleotides) were identified, and the codons were removed from all three species. Synonymous (d*S*) substitution rates, nonsynonymous (d*N*) substitution rates, and d*N*/d*S* ratios were estimated for X chromosomes and autosomes separately in both *S. mimosarum* and *S. africanus* using PAML ver. 4.6 (Yang 2007). 95% confidence limits of d*N*, d*S*, and d*N*/d*S* were estimated by bootstrapping over the genes (*n* = 1,000) and producing one overall (across genes) estimate of d*N*, d*S*, and d*N*/d*S* for each sampling.

#### Molecular Diversity at X Chromosomes and Autosomes

A RAD reference was constructed for both *S. mimosarum* and *S. africanus* in two steps. First, all sequences represented by at least three identical reads were obtained using a custom program in all individuals separately (“clc_find_maximal,” see Supplementary Material online for more information). In the second step, species-specific RAD references were created by grouping all the resulting sequences from conspecific individuals with >98% similarity using a custom program (“clc_find_groups,” for more detail, see Supplementary Material online). The resulting RAD reference sets were mapped to the genome sequence of *S. mimosarum* (Sanggaard et al. 2014), and in cases with more than one reference sequence mapped to same position, all but one were removed using a custom script (“remove_dup.tcsh,” for more detail, see Supplementary Material online), giving two final RAD reference sets, one for each species. Each individual was subsequently mapped to these RAD reference sets using CLC Genomics Workbench 7, and consensus sequences were extracted from all mappings having a minimum of 8 reads and a maximum of 40 reads. Ambiguous bases (IUPAC) were called when the least frequent base was supported by at least three reads and/or if its proportion was >10%, but also considering the read quality score as implemented in the CLC Genomics Workbench 7. If a consensus sequence had >2.5% ambiguous bases it was discarded assuming that the mapped reads originated from more than one genomic position. For each consensus sequence, we called two alleles that were subsequently aligned per locus for all individuals within each population using PRANK (Loytynoja and Goldman 2008). We discarded alignments with less than five individuals represented, and separated remaining alignments into X chromosome and autosome based on the mapping of the RAD reference sets to the *S. mimosarum* genome scaffolds (see above). Since *S. africanus* is closely related to *S. mimosarum*, we interpreted the *S. africanus* RADs mapping to *S. mimosarum* X chromosome scaffolds as X chromosomes in *S. africanus* as well. For each set of alignments, all the alignments were concatenated, with missing sequences written as gaps, and split into equally long subalignments. This length was identical across all populations, X chromosome sets and autosome sets. This universal length of the subalignments was set such that the population with smallest X chromosome coverage had 15 subalignments (KRU: 136,432 bp). Within population genetic diversity (Tajima 1983) was calculated for each subalignment using the package ape in R (Paradis et al. 2004; R Core Team 2015), and average pi was calculated for X chromosomes and autosomes for each population. 95% confidence limits were estimated by bootstrapping over the sub alignments (*n* = 10,000).

#### McDonald–Kreitman Test

Rad sequence data from all *S. mimosarum* individuals were mapped to the reference genome using bwa (Li and Durbin 2009). Polymorphic positions were called in positions with minimum coverage of 10× using Samtools and bcftools (Li et al. 2009). Sites that were polymorphic in the RAD sequence data and sites that differed between RAD sequence data and the reference genome were considered. snpEff was used to identify variants located in protein coding positions, and if they were synonymous or nonsynonymous (Cingolani et al. 2012). The number of synonymous and nonsynonymous substitutions on X chromosomes and autosomes were taken from PAML analyses described earlier.

#### Variation in Diversity between X Chromosomes and Autosomes

Alignments of RAD loci from separate scaffolds were concatenated, and pi was estimated per scaffold with three or more RAD loci using the package ape (Paradis et al. 2004) in R (R Core Team 2016). Coefficient of variation was estimated for autosome and X chromosome scaffolds separately for each population using the estimator SD/average.

#### Simulations

We simulated DNA sequences under a recurrent bottleneck scenario using fastsimcoal2 (Excoffier et al. 2013). This was done with two different population sizes, 20,000 and 15,000 representing autosomes and X chromosomes, respectively, and using a mutation rate of 1.2E-8. These parameters were chosen to reach diversity similar to the estimates obtained from the RAD sequence data. Data were simulated to mimic our RAD sequence data by simulating 20,000 independent loci of 100 bp. Data were simulated under four different bottlenecks scenarios; 50 and 100 generations between bottlenecks combined with magnitude of bottlenecks of 1% and 10% (supplementary fig. 9, Supplementary Material online). Pi was estimated at different time points using the package ape (Paradis et al. 2004) in R (R Core Team 2016).

#### Statistical Analyses

When testing for species differences in piX/piA it was necessary to account for populations using a random effect in a mixed model in the package lme4 (Bates et al. 2015) in R (R Core Team 2016). Data used for this test were the subalignments of equal length created for the bootstrapping (see above). For each population, we grouped the autosomal subalignments in as many groups as there were subalignments of the X chromosomes. We then calculated the median (due to a highly skewed distribution of pi in *S. mimosarum* which has high frequency of scaffolds with zero pi) pi for each autosomal group and paired it randomly with an X chromosome subalignment, in this way, we got several independent estimates of piX/piA for each population. The statistical significance of the effect of species was assessed using a likelihood ratio test. To further test for population differentiation within species, we constructed a linear model for each species, containing population as the only predictor variable, and used *F*-tests and Tukey’s HSD method for post hoc comparisons. To test for differences in d*N*/d*S* between autosomes and X chromosomes within a species, we used a randomization test in which we permuted chromosomal origin (autosome or X chromosome) of all the genes (*n* permutations = 1,000) and estimated one overall (across genes) estimate of d*N*/d*S* for genes assigned to autosome or X chromosome in each sampling. We then estimated X_(d__*N*__/d__*S*__)_−A_(d__*N*__/d__*S*__)_ for each permutation, and used this as a test-statistic in a two-tailed test by comparison to the observed difference. We used the same approach when testing for differences in d*S*, but here we used the ratio d*S*_X_/d*S*_A_ as a test-statistic. A similar approach was used to test for differences in A_(d__*N*__/d__*S*__)_ or in X_(d__*N*__/d__*S*__)_ between the two species, except here we permuted the species origin of each gene and used the test-statistics A_africanus (d__*N*__/d__*S*__)_−A_mimosarum (d__*N*__/d__*S*__)-_ and X_africanus (d__*N*__/d__*S*__)_−X_mimosarum (d__*N*__/d__*S*__)_. Finally, when analyzing whether the difference in d*N*/d*S* between X-linked and autosomal genes differs between S. *mimosarum* and *S. africanus*, we used (X_(d__*N*__/d__*S*__)_/A_(d__*N*__/d__*S*__)_)_africanus_−(X_(d__*N*__/d__*S*__)_/A_(d__*N*__/d__*S*__)_)_mimosarum_ as a test-statistic.

##### X Chromosome Mutation Rate

We estimated the male-to-female mutation rate from synonymous divergence under the assumption that the synonymous mutation rate equals the synonymous substitution rate (Kimura 1983), by comparing synonymous divergence at X chromosomes and autosomes. We used the formula *k*_x_/*k*_A_ = (2/3)(2+α)/(1+α) following Miyata et al. (1987), where k is the synonymous sequence divergence. We obtained two independent estimates; 1) synonymous divergence (d*S*) between the two species estimated from transcriptome data, and 2) divergence between the two genetically isolated groups of *S. mimosarum* populations from Madagascar and South Africa, respectively, estimated from RAD data. The latter analysis was done under the assumption that RAD loci evolve neutrally. To adjust for different times to accumulate substitutions for X chromosomes and autosomes due to different coalescence times of X chromosomes and autosomes in the ancestral species/population caused by differences in Ne, we used coalescence calculations to adjust *k*_x_/*k*_A_. These calculations are based on coalescence times in the ancestral species/population, which is a function of effective ancestral population sizes (*N*_A_). Approximate *N*_A_ estimates were obtained from Settepani et al. (2017).

## Supplementary Material

Supplementary data are available at *Molecular Biology and Evolution* online.

## Supplementary Material

Supplementary_Material_msz074
