# Supplementary material for: Evidence for Faster X Chromosome Evolution in Spiders
Source: Mol Biol Evol. 2019 Mar 26;36(6):1281–93. doi: 10.1093/molbev/msz074 (PMC6526907; doi:10.1093/molbev/msz074)
Supplement: Supplementary_Material_msz074 [file supplementary_material_msz074.zip › supplementary fig. 9.pdf]

Autosome: N = 16,000
Sex chromosomes: N = 12,000
Mutation rate: 0.000000012
loci: 20,000 (100bp)
Transition/transversion: 0.33

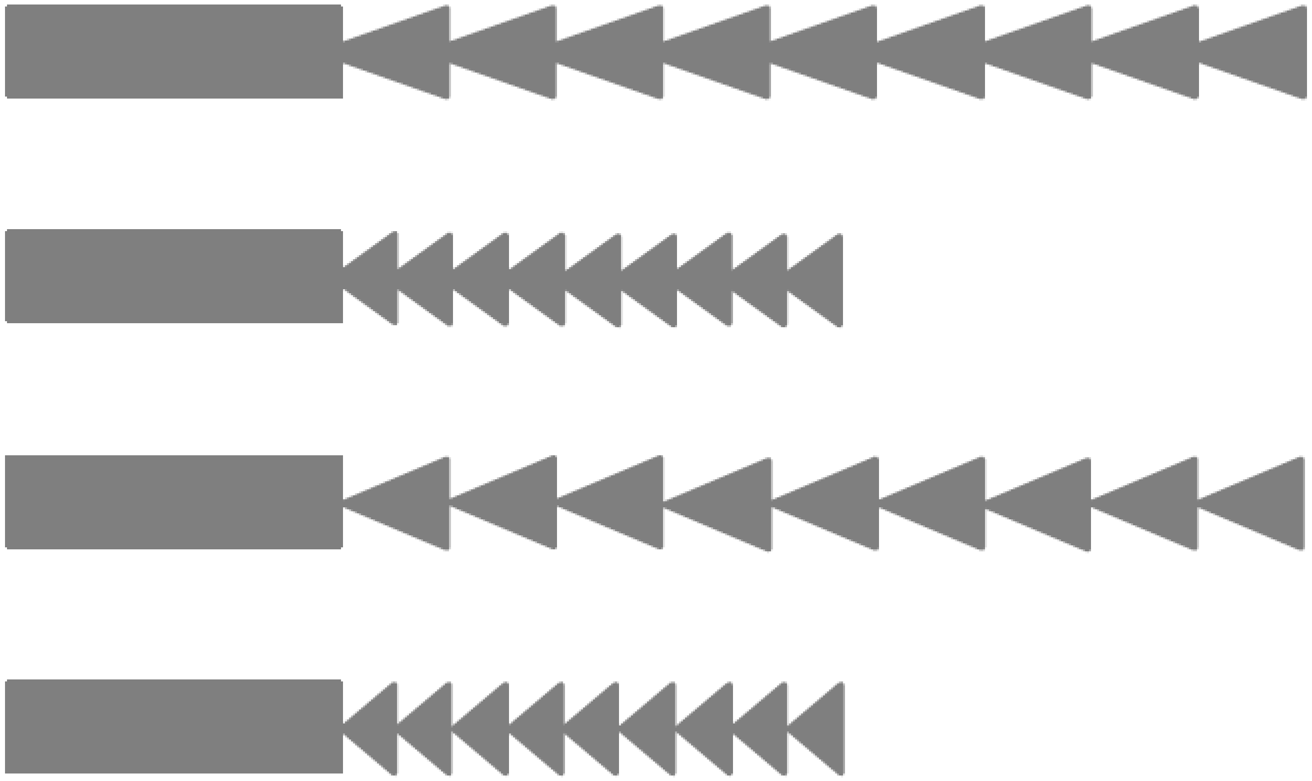

Bottleneck:
- 100th generation
- 10% resize

Bottleneck:
- 50th generation
- 10% resize

Bottleneck:
- 100th generation
- 1% resize

Bottleneck:
- 50th generation
- 1% resize
