# Supplementary material for: Evidence for Faster X Chromosome Evolution in Spiders
Source: Mol Biol Evol. 2019 Mar 26;36(6):1281–93. doi: 10.1093/molbev/msz074 (PMC6526907; doi:10.1093/molbev/msz074)
Supplement: Supplementary_Material_msz074 [file supplementary_material_msz074.zip › supplementary Materials.docx]

***Supplementary material accompanying:***

**Faster X chromosome evolution in spiders**

Jesper Bechsgaard^1*^, Mads Fristrup Schou^1*^, Bram Vanthournout^1,2^, Frederik Hendrickx^3,4^, Bjarne Knudsen^5^, Virginia Settepani^1^, Mikkel Heide Schierup^1,6^, Trine Bilde^1^

*^1^ Department of Bioscience, Aarhus University, 8000 Aarhus C, Denmark*

*^2^ Evolution and Optics of Nanostructure group (EON), Biology Department, Ghent University, Ghent, Belgium*

*^3^ Royal Belgian Institute of Natural Sciences, Brussels, Belgium*

*^4^ Terrestrial Ecology Unit (TEREC), Biology Department, Ghent University, Ghent, Belgium*

*^5^ Qiagen Bioinformatics, Aarhus, Denmark*

*^6^ Bioinformatics Research Centre (BiRC), Aarhus University, 8000 Aarhus C, Denmark*

**These authors contributed equally*

*Corresponding author: Trine Bilde, email: Trine.Bilde@bios.au.dk*

***Table of contents***

1. ***Bayesian Mixture analysis on proportions (logits)***
2. ***Making a RAD reference, mapping and variant calling***
3. ***Figure legends of supplementary figures***
4. ***Table legends of supplementary tables***
5. ***Bayesian Mixture analysis on proportions (logits)***

*Theory*

Let *p*_0,_*_i_* represent the proportion of Sample 0 reads that map to scaffold *i*.

The number of reads in Sample 0 on scaffold *i* (*N_0,i_*) follows binomial distribution, with parameters total number of reads (*N_tot,i_*) and proportion *p*_0,_*_i_*,

$$N_{0,i}\sim B\left( p_{0,i},N_{tot,i} \right)$$

The distribution of the proportion of reads on Sample 0, *p*_0,_*_i_* , across scaffolds is assumed to be a mixture of *p*_0_ = 0 (reads mapping to scaffolds on the X1X2 chromosomes) and *p*_0_ = 0.5 (reads mapping to scaffolds on the autosomes).

However, because of errors in the sorting process, the means of both distributions do not equal these values, but need to be estimated from the data.

We assume that the logit of *p*_0,_*_i_*, ln(*p*_0,_*_i_* /1- *p*_0,_*_i_*) is distributed as a sum of *k* = 2 normal distributions (mixture), each with proportion *P_k_*

$$logit\left( p_{0,i} \right)\sim\sum_{k=1}^{2} P_{k}N(\mu_{0,k},\sigma_{0,k})$$

Hence, *P*_1_ estimates the proportion of scaffolds on the autosomes, *µ*_0,1_ estimates the mean of the (logits of) proportions of Sample 0 reads mapping to the autosomes and *σ*_0,1_, estimates the standard deviation of the distribution the (logits of) proportions of reads mapping to the autosomes.

*P*_2_ estimates the proportion of scaffolds on the X chromosomes, *µ*_0,2_ estimates the mean of the (logits of) proportions of Sample 0 reads mapping to the X chromosomes and *σ*_0,2_, estimates the standard deviation of the distribution the (logit) proportions of reads mapping to the X chromosomes.

It is necessary to work with logits, as proportions - which are bound between 0 and 1 - do not follow a normal distribution. This is particularly the case when the mean proportion is close to 0, which is the case for the reads that map to the X chromosomes. Logits of proportions in contrast, are expected to follow a normal distribution. Estimates of µ can easily be transformed to proportions by taking the expit: exp(µ) = exp(µ)/(1+exp(µ)).

*Results*

The analysis shows that a proportion of P_1_= 0.9047 that are scaffolds are located on the autosomes and a proportion P_2_ = 0.0953 of the scaffolds are located on the X chromosomes. The logit of the proportion of Sample 0 reads that map to the autosomes is 0.0002813. Transformation to obtain the proportion results in *p*_reads,autosomes_=0.50007, which is very close to the expectation of 0.5. The logit of the proportion of Sample 0 reads of scaffolds that map to the X chromosomes equals -2.005. Transformation reveals that *p*_reads,X chromosomes_ = 0.119.

Table 1:

| **Node** | **Mean** | **sd** | **MC error** | **2.5%** | **Median** | **97.5%** | **Start** | **Sample** |
| --- | --- | --- | --- | --- | --- | --- | --- | --- |
| P_1_ | 0.9047 | 0.005718 | 2.966E-4 | 0.8927 | 0.9049 | 0.915 | 1001 | 4000 |
| P_2_ | 0.0953 | 0.005718 | 2.966E-4 | 0.08496 | 0.09509 | 0.1074 | 1001 | 4000 |
| *µ*_0,1_ | 2.813E-4 | 0.00853 | 2.349E-4 | -0.01647 | 2.619E-4 | 0.01669 | 1001 | 4000 |
| *µ*_0,2_ | -2.005 | 0.04872 | 0.00367 | -2.103 | -2.004 | -1.91 | 1001 | 4000 |
| *σ*_0,1_ | 0.4002 | 0.007056 | 1.962E-4 | 0.3863 | 0.4003 | 0.4143 | 1001 | 4000 |
| *σ*_0,2_ | 1.649 | 0.1698 | 0.01351 | 1.282 | 1.655 | 1.968 | 1001 | 4000 |

To test whether the assumed model agrees with the data, we plotted the observed logits of the proportions against the estimated distribution. Although the model agrees reasonably well with the observed data, there is still some mismatch that is likely due to the logits of the proportions deviating from a normal distribution. Based on the model, one could put high confidence that scaffolds with a maximum logit of -1 (corresponding to *p*_0,i_ ≤ 0.27) are on the X chromosomes. If compared to the observed values, a more stringent value should be used (e.g. -1.5 or -2, corresponding to *p*_0,i_ ≤ 0.18 and *p*_0,i_ ≤ 0.12 respectively). Cut-off values of -2, -1.5 and -1 will contain 50%, 62% and 73% of the scaffolds located on the X chromosomes.

1. ***Making a RAD reference, mapping and variant calling***

For each species, reads from all individuals were grouped based on similarity (98%), and we extracted a consensus sequence for each group. Indel polymorphism was allowed in the grouping process. The consensus sequences represent the RAD reference of the given species.

The RAD reference sequences were mapped to the genome of *S. mimosarum* to divide the RAD reference sequences into X chromosome RADs and autosome RADs. Some RAD reference sequences mapped to the same location in the genome. This can happen if two regions of the genome are very similar, but one of them not represented in the published genome sequence or if a region has so high diversity that different alleles will form two groups and therefore two consensus sequences. Whenever two or more RADs mapped to the same location in the genome, all but one was removed, thereby reducing the RAD reference to sequences that all map to different genomic locations.

The reads of each individual were mapped to the RAD reference sequenced using ‘Maps Reads to Reference’ in CLC genomics 7.5.1 (default parameters), and variants were called using the ‘Fixed Ploidy Variant Detection’ in RADs with coverage between 10x and 40x in CLC genomics 7.5.1 (default parameters).

1. ***Figure legends of supplementary figures***

*Supplementary figure S1.*

Observed and fitted proportions of reads mapped to Sample 0 (P_0_) in each scaffold. Data is presented both in logit (top graph) and raw (lower graph). Black bars represent the observed results while the fitted lines were calculated using Bayesian mixture analysis.

*Supplementary figure S2.*

Codon usage bias for autosomes and X chromosomes in *S. mimosarum* and *S. africanus*. Randomization tests showed no difference between autosomes and X chromosomes in *S. mimosarum* (*P* = 1.00) or *S. africanus* (*P* = 1.00), and no difference across species in autosomes (*P* = 0.774) and X chromosomes (*P* = 0.814). Error bars represent 95% confidence limits obtained by bootstrapping.

*Supplementary figure S3.*

Population specific distribution of pi in scaffolds of S. mimosarum and S. africanus for both autosomes and X chromosomes.

*Supplementary figure S4.*

Simulated effect of four different recurrent bottlenecks scenarios and a constant population size scenario on the X chromosome to autosome diversity ratio. Recurrent bottlenecks were simulated either with 50 or 100 generations between bottlenecks and with the magnitude of bottlenecks being either 1% or 10%. See also supplementary figure S6.

*Supplementary figure S5.*

Schematic presentation of the divergence (RAD data) of X chromosomes and autosomes in *S. mimosarum* from Madagascar and South Africa as well as the synonymous divergence (RNA sequencing data) of X chromosomes and autosomes between *S. mimosarum* and *S. africanus*. We calculated the expected dRAD_X_/dRAD_A_ by assuming an ancestral population size (N_A_) of 20,000, sex ratio of 1:1, and species split time of 20,000 years. Under the assumptions of N_A_ equal 300,000, sex ratio of 1:1, and species split time of 1 million years, we calculated a predicted dS_X_/dS_A_ for comparison with the observed estimates.

*Supplementary figure S6.*

The left grey bar shows the predicted neutral X to A diversity ratio with an operational sex ratio of 1:1, equal mutation rate on X chromosomes and autosomes, and constant population size (‘null prediction’). The second grey bar shows the observed X to A diversity ratio in *S. africanus* that we infer is lower than predicted due to a lower mutation rate and/or more hitchhiking events on X chromosomes compared to autosomes. The last two grey bars show the observed X to A diversity ratio in *S. mimosarum* that we infer has originated under two possible alternative evolutionary histories. The first possibility is that the same evolutionary forces as in *S. africanus* (a lower mutation rate and/or more hitchhiking events on X chromosomes compared to autosomes) have caused a lower X to A diversity ratio compared to the null prediction, but that female bias has caused an elevated X to A diversity ratio. The second possibility is that the effect of female bias is even stronger, but that fluctuating population sizes operates in the opposite direction, and cause a decreased X to A diversity ratio. The light grey bar sketches the effect of fluctuating population sizes and female bias. Vertical red lines show observed 95% CI.

*Supplementary figure S7.*

The minimum P_0_ cut-off for a scaffold to be considered putatively autosomal affects the number of autosomal scaffolds used for analyses. We investigated the consequence of three different cut-offs on resulting estimates of genetic diversity from RAD-seq data across the populations in the two species. For the full analyses in the main document we choose to use a cut-off at P_0_ = 0.3 (false discovery rate of 2.5%).

*Supplementary figure S8.*

The minimum P_0_ cut-off for a scaffold to be considered putatively autosomal affects the number of autosomal scaffolds used for analyses. We investigated the consequence of three different cut-offs on resulting estimates of dN/dS from RNA-seq data across the populations in the two species. For the full analyses in the main document we choose to use a cut-off at P_0_ = 0.3 (false discovery rate of 2.5%).

*Supplementary figure S9.*

Parameter settings for simulations of recurrent bottlenecks. Recurrent bottlenecks were simulated either with 50 or 100 generations between bottlenecks and with the magnitude of bottlenecks being either 1% or 10%.

1. ***Table legends of supplementary tables***

*Supplementary table S1.*

Docx file. Summary of results found in previous studies contrasting substitution patterns and diversity on X chromosomes and autosomes to investigate faster-X evolution.

*Supplementary table S2.*

Xlsx file. Contains parameters of the two overlapping distributions of *P*_0_ and a formula for calculating the false discovery rate of assignment of scaffolds as either autosome or X chromosomes.

*Supplementary table S3.*

Txt file. *S. mimosarum* scaffolds assigned as putatively X chromosomal or autosomal. Scaffold length is also provided in the file.

*Supplementary table S4.*

Docx file. Descriptive data summarizing scaffolds and genes assigned as putatively autosomal or belonging to the X chromosomes.

*Supplementary table S5.*

Txt file. *S. mimosarum* genes and their putatively scaffold type (autosome or X chromosome).
