# Supplementary material for: Evidence for Faster X Chromosome Evolution in Spiders
Source: Mol Biol Evol. 2019 Mar 26;36(6):1281–93. doi: 10.1093/molbev/msz074 (PMC6526907; doi:10.1093/molbev/msz074)
Supplement: Supplementary_Material_msz074 [file supplementary_material_msz074.zip › supplementary table 1.docx]

**Table S1**. Summary of results found in previous studies contrasting substitution patterns and diversity on X chromosomes and autosomes to investigate faster-X evolution.

| Species | Faster-X | Suggested cause | Reference |
| --- | --- | --- | --- |
| Mammals | Yes | Positive selection | (Torgerson and Singh 2006) |
| Rabbits | Yes/no | Positive selection | (Carneiro, et al. 2012) |
| Mouse | Yes | Positive selection | (Baines and Harr 2007) |
| Humans | Yes | Purifying/positive selection | (Arbiza, et al. 2014) |
| Humans | Yes | Positive selection | (Hammer, et al. 2010) |
| Primates (human and chimpanzee) | Yes | Positive selection | (Lu and Wu 2005) |
| Primates (human, orangutan, rhesus, marmoset) | Yes/no | Lineage specific | (Xu, et al. 2012) |
| Primates (chimpanzee) | Yes | Positive selection and purifying selection | (Hvilsom, et al. 2012) |
| Zebra finch and chicken | Yes | - | (Mank, et al. 2007) |
| Zebra finch and chicken | Yes | Drift/smaller N_e_ | (Mank, et al. 2010) |
| Fowls | Yes | Promiscuity and sexual selection | (Wright, et al. 2015) |
| Aphids | Yes | Relaxed selection | (Jaquiery, et al. 2018) |
| Butterflies | No | Purifying selection | (Rousselle, et al. 2016) |
| Silkmoth | Yes | Positive selection | (Sackton, et al. 2014) |
| *Drosophila melanogaster* and *D. simulans* | No | - | (Betancourt, et al. 2002) |
| *D. melanogaster* and *D. simulans* | Yes | Positive selection | (Hu, et al. 2013) |
| *D. miranda*, *D. melanogaster*, *D. yakuba* and *D. pseudoobscura* | No | Lack of partially recessive beneficial mutations | (Thornton, et al. 2006) |
| *D. miranda*, *D. melanogaster*, *D. yakuba* and *D. pseudoobscura* | Yes/no | Contrast specific | (Counterman, et al. 2004) |

**References**

Arbiza L, Gottipati S, Siepel A, Keinan A. 2014. Contrasting x-linked and autosomal diversity across 14 human populations. American Journal of Human Genetics 94:827-844.

Baines JF, Harr B. 2007. Reduced X-linked diversity in derived populations of house mice. Genetics 175:1911-1921.

Betancourt AJ, Presgraves DC, Swanson WJ. 2002. A test for faster X evolution in *Drosophila*. Molecular Biology and Evolution 19:1816-1819.

Carneiro M, Albert FW, Melo-Ferreira J, Galtier N, Gayral P, Blanco-Aguiar JA, Villafuerte R, Nachman MW, Ferrand N. 2012. Evidence for widespread positive and purifying selection across the european rabbit (*Oryctolagus cuniculus*) Genome. Molecular Biology and Evolution 29:1837-1849.

Counterman BA, Ortiz-Barrientos D, Noor MAF. 2004. Using comparative genomic data to test for fast-x evolution. Evolution 58:656-660.

Hammer MF, Woerner AE, Mendez FL, Watkins JC, Cox MP, Wall JD. 2010. The ratio of human X chromosome to autosome diversity is positively correlated with genetic distance from genes. Nature Genetics 42:830-831.

Hu TT, Eisen MB, Thornton KR, Andolfatto P. 2013. A second-generation assembly of the *Drosophila* simulans genome provides new insights into patterns of lineage-specific divergence. Genome Research 23:89-98.

Hvilsom C, Qian Y, Bataillon T, Li YR, Mailund T, Salle B, Carlsen F, Li RQ, Zheng HC, Jiang T, et al. 2012. Extensive X-linked adaptive evolution in central chimpanzees. Proceedings of the National Academy of Sciences of the United States of America 109:2054-2059.

Jaquiery J, Stoeckel S, Rispe C, Mieuzet L, Legeai F, Simon JC. 2012. Accelerated evolution of sex chromosomes in aphids, an X0 system. Molecular Biology and Evolution 29:837-847.

Lu J, Wu CI. 2005. Weak selection revealed by the whole-genome comparison of the X chromosome and autosomes of human and chimpanzee. Proceedings of the National Academy of Sciences of the United States of America 102:4063-4067.

Mank JE, Axelsson E, Ellegren H. 2007. Fast-X on the Z: Rapid evolution of sex-linked genes in birds. Genome Research 17:618-624.

Mank JE, Nam K, Ellegren H. 2010. Faster-Z evolution is predominantly due to genetic drift. Molecular Biology and Evolution 27:661-670.

Rousselle M, Faivre N, Ballenghien M, Galtier N, BNabholz B. 2016. Hemizygosity enhances purifying selection: lack of fast-Z evolution in two satyrine butterflies. Genome Biology and Evolution 8:12.

Sackton TB, Corbett-Detig RB, Nagaraju J, Vaishna L, Arunkumar KP, Hartl DL. 2014. Positive selection drives faster-Z evolution in silkmoths. Evolution 68:2331-2342.

Thornton K, Bachtrog D, Andolfatto P. 2006. X chromosomes and autosomes evolve at similar rates in *Drosophila*: No evidence for faster-X protein evolution. Genome Research 16:498-504.

Torgerson DG, Singh RS. 2006. Enhanced adaptive evolution of sperm-expressed genes on the mammalian X chromosome. Heredity 96:39-44.

Wright AE, Harrison PW, Zimmer F, Montgomery SH, Pointer MA, Mank JE. 2015. Variation in promiscuity and sexual selection drives avian rate of Faster-Z evolution. Molecular Ecology 24:1218-1235.

Xu K, Oh S, Park T, Presgraves DC, Yi SV. 2012. Lineage-specific variation in slow- and fast-X evolution in primates. Evolution 66:1751-1761.

Jaquiery J, Peccoud J, Ouisse T, Legeai F, Prunier-Leterme N, Gouin A, Nouhaud P, Brisson JA, Bickel R, Purandare S, et al. 2018. Disentangling the causes for faster-X evolution in aphids. Genome Biology and Evolution 10:507-520.
