# Supplementary material for: Evidence for Faster X Chromosome Evolution in Spiders
Source: Mol Biol Evol. 2019 Mar 26;36(6):1281–93. doi: 10.1093/molbev/msz074 (PMC6526907; doi:10.1093/molbev/msz074)
Supplement: Supplementary_Material_msz074 [file supplementary_material_msz074.zip › supplementary table 4.docx]

**Table S4**. Descriptive data summarizing scaffolds and genes assigned as putatively autosomal or belonging to the X-chromosomes.

|  | Autosomes | | Sex chromosomes | |
| --- | --- | --- | --- | --- |
| Median scaffold length | 408,754 | | 471,004 | |
| Total bp covered | 1,772,262,647 | | 246,725,799 | |
| Number of scaffolds | 3,653 | | 450 | |
| Number of genes | 17,103 | | 2132 | |
| Number of genes analysed | 4,641 | | 523 | |
| Estimated based on flowcytometry | 85% | | 15% | |
|  | *S. mimosarum* | *S. africanus* | *S. mimosarum* | *S. africanus* |
| Total number scaffolds covered by rad loci post filtering | 2,525 | 2,770 | 355 | 336 |
