# Supplementary figures and images for: Evidence for Faster X Chromosome Evolution in Spiders
Source: Mol Biol Evol. 2019 Mar 26;36(6):1281–93. doi: 10.1093/molbev/msz074 (PMC6526907; doi:10.1093/molbev/msz074)

**Observed and fitted logit(P reads in Sample 0)**

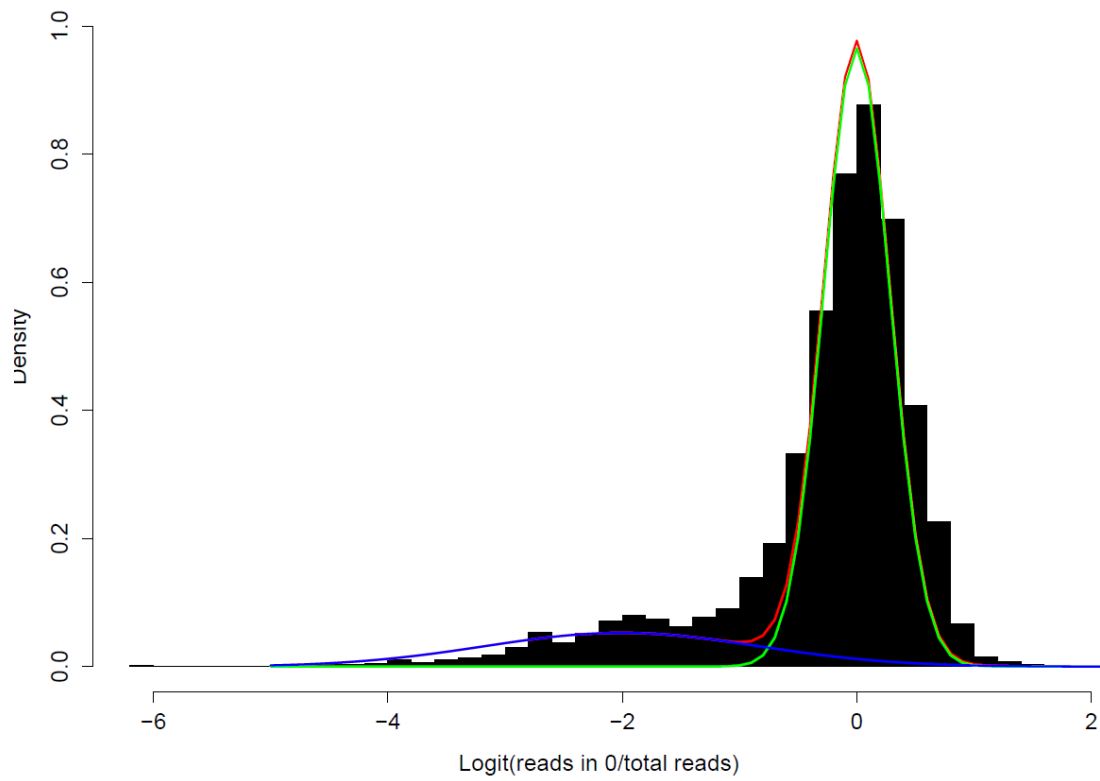

**Observed and fitted P reads in Sample0**

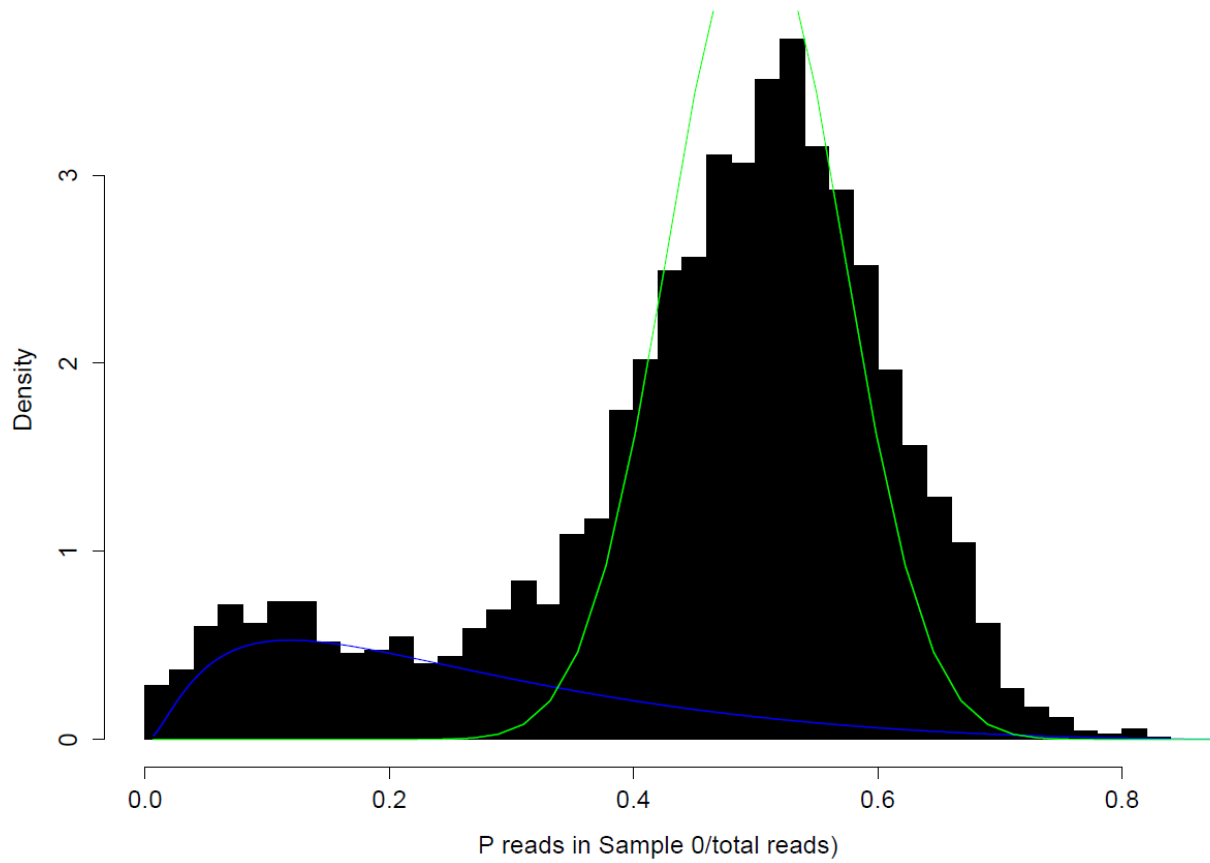

Supplement: Supplementary_Material_msz074 [file supplementary_material_msz074.zip › supplementary fig. 1.pdf]

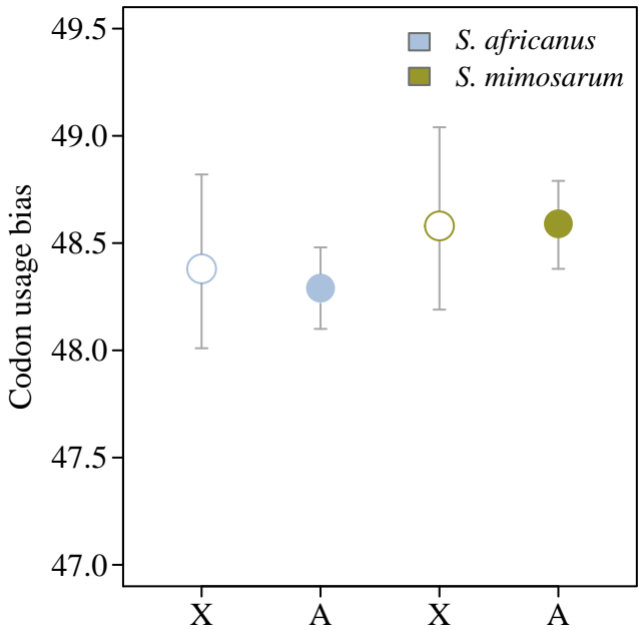

Supplement: Supplementary_Material_msz074 [file supplementary_material_msz074.zip › supplementary fig. 2.pdf]

# *S. mimosarum*

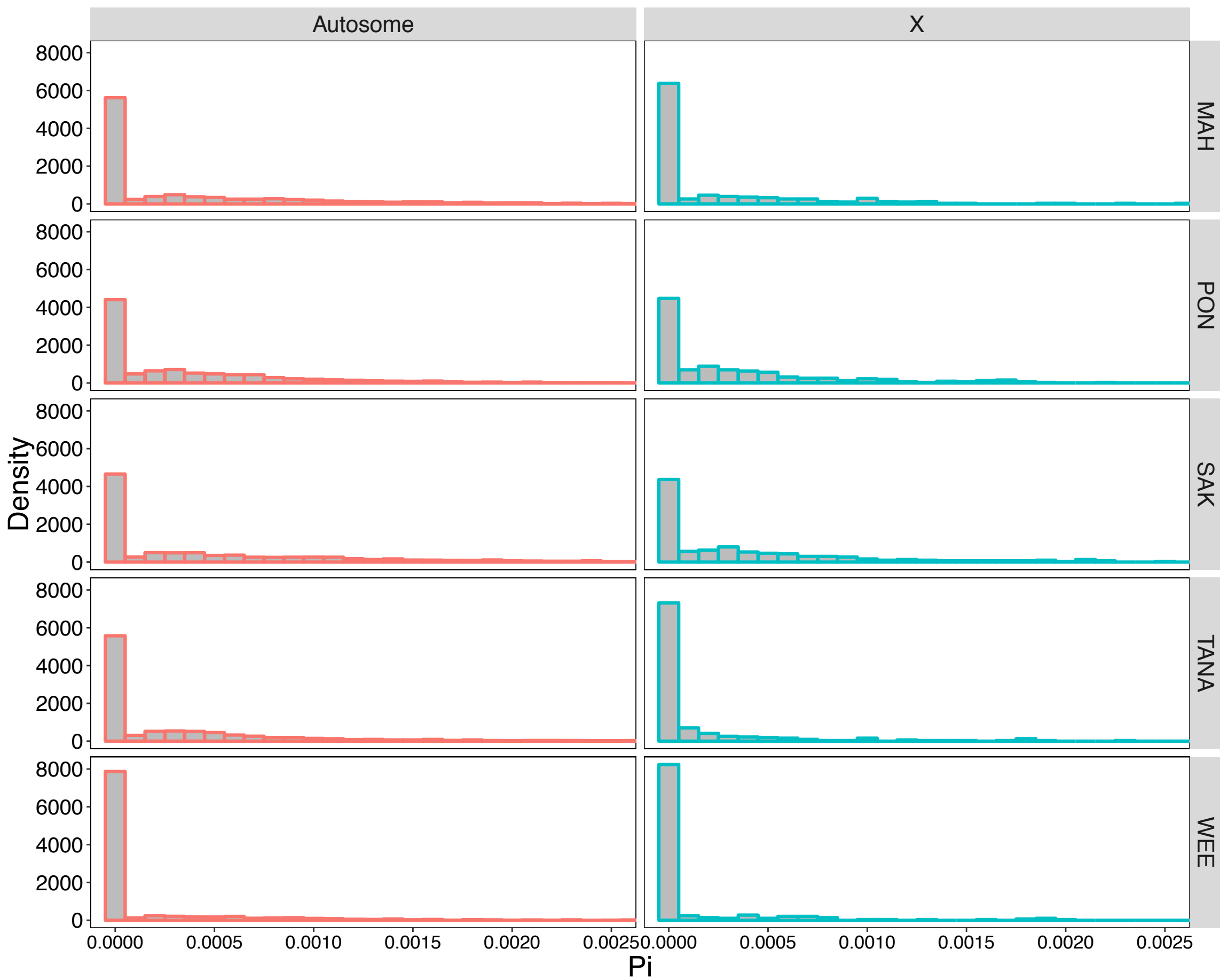

# *S. africanus*

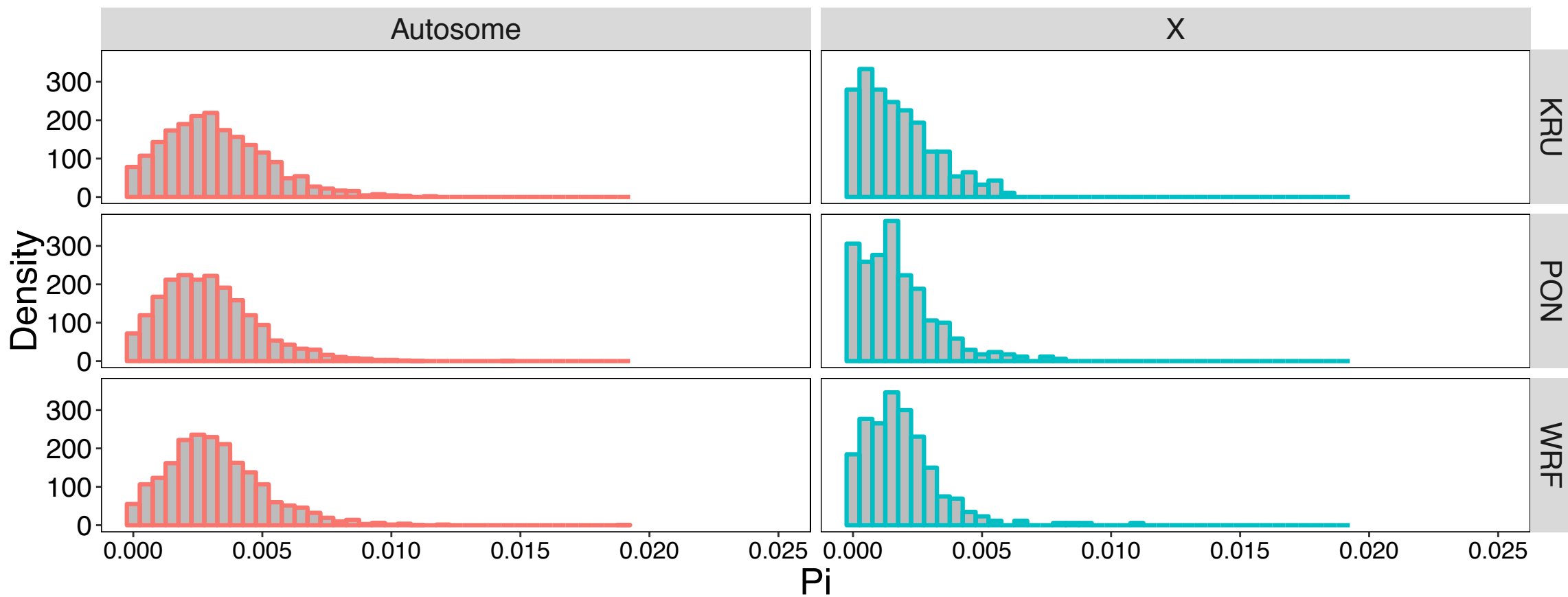

Supplement: Supplementary_Material_msz074 [file supplementary_material_msz074.zip › supplementary fig. 3.pdf]

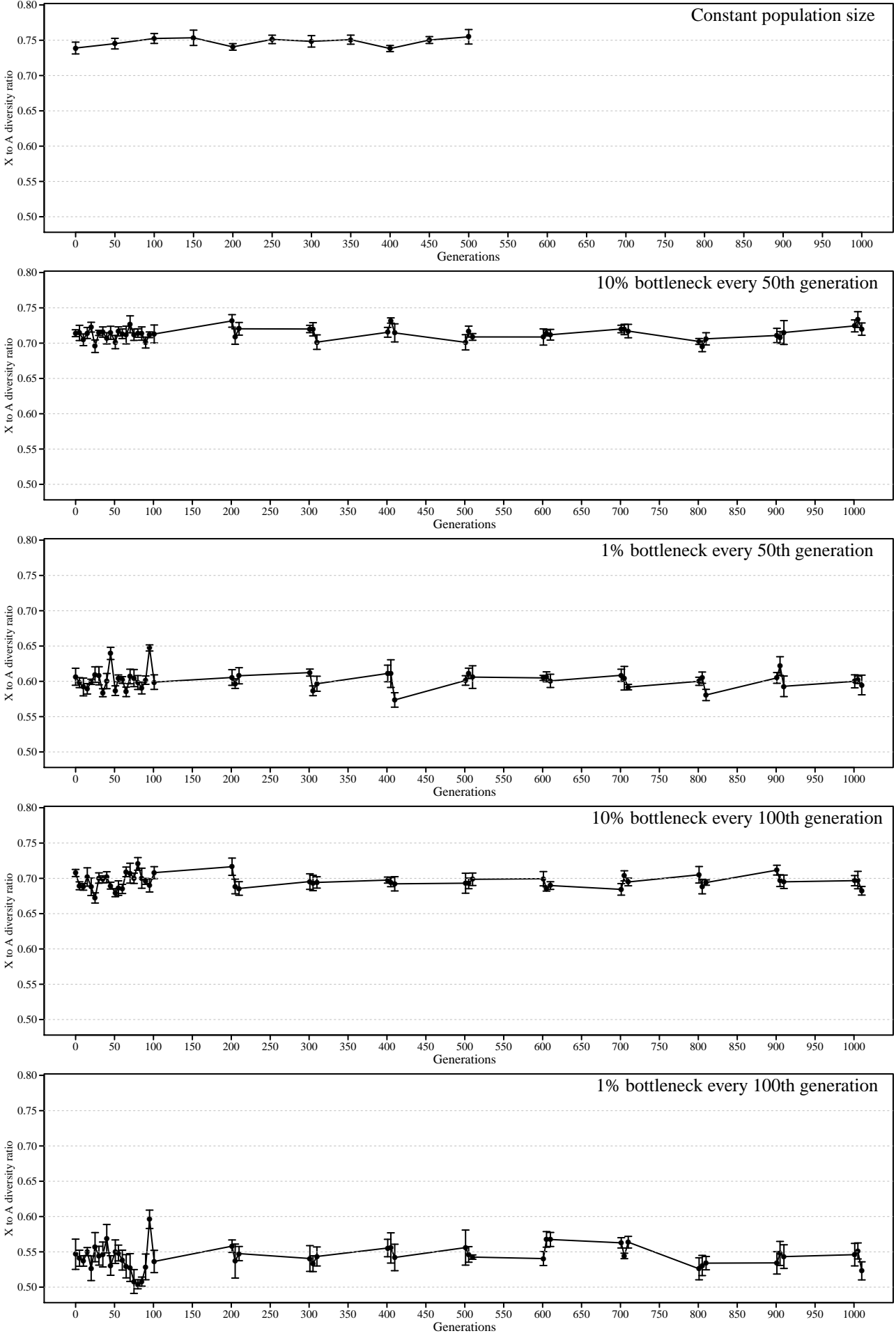

Supplement: Supplementary_Material_msz074 [file supplementary_material_msz074.zip › supplementary fig. 4.pdf]

## RAD divergence in *S. mimosarum*

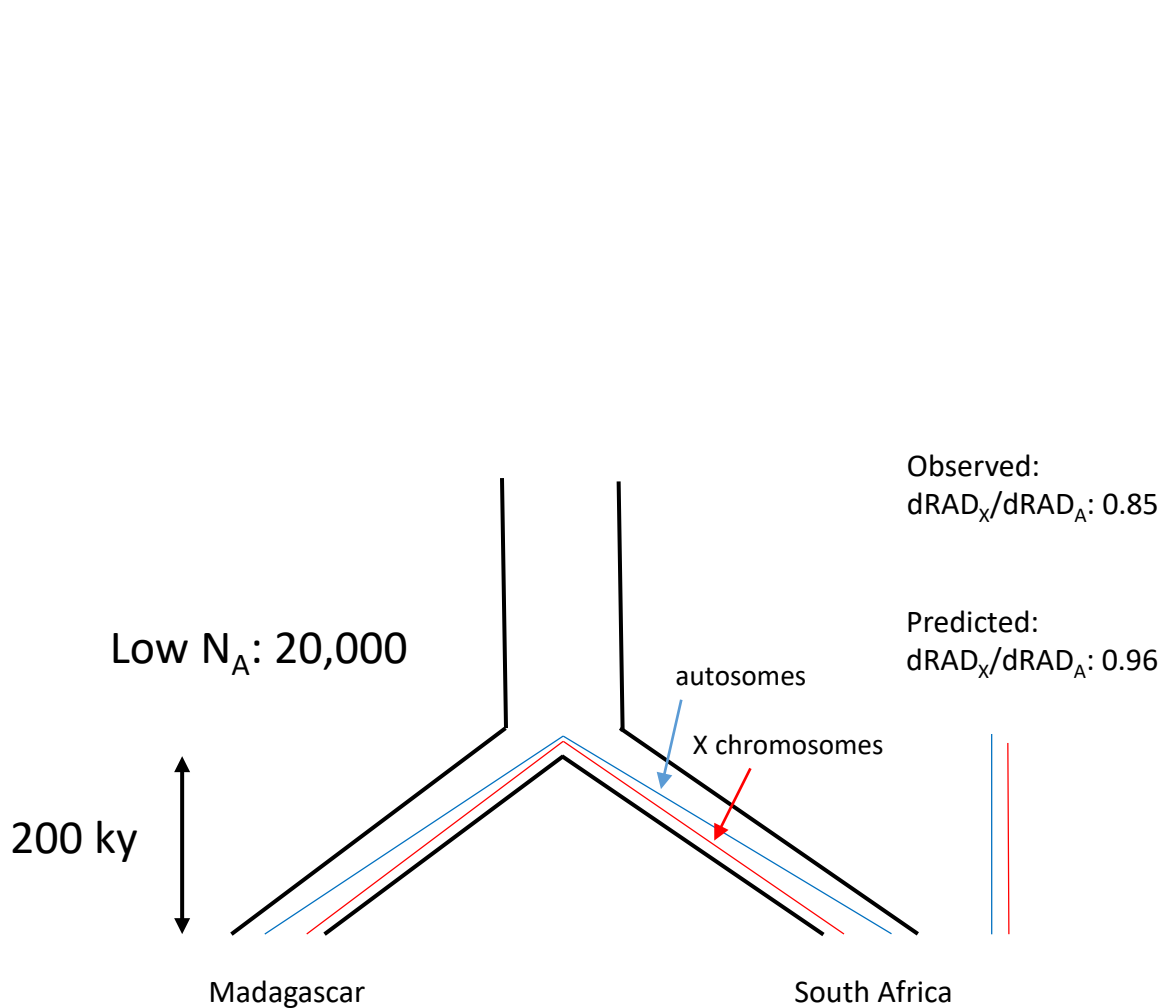

## Synonymous divergence

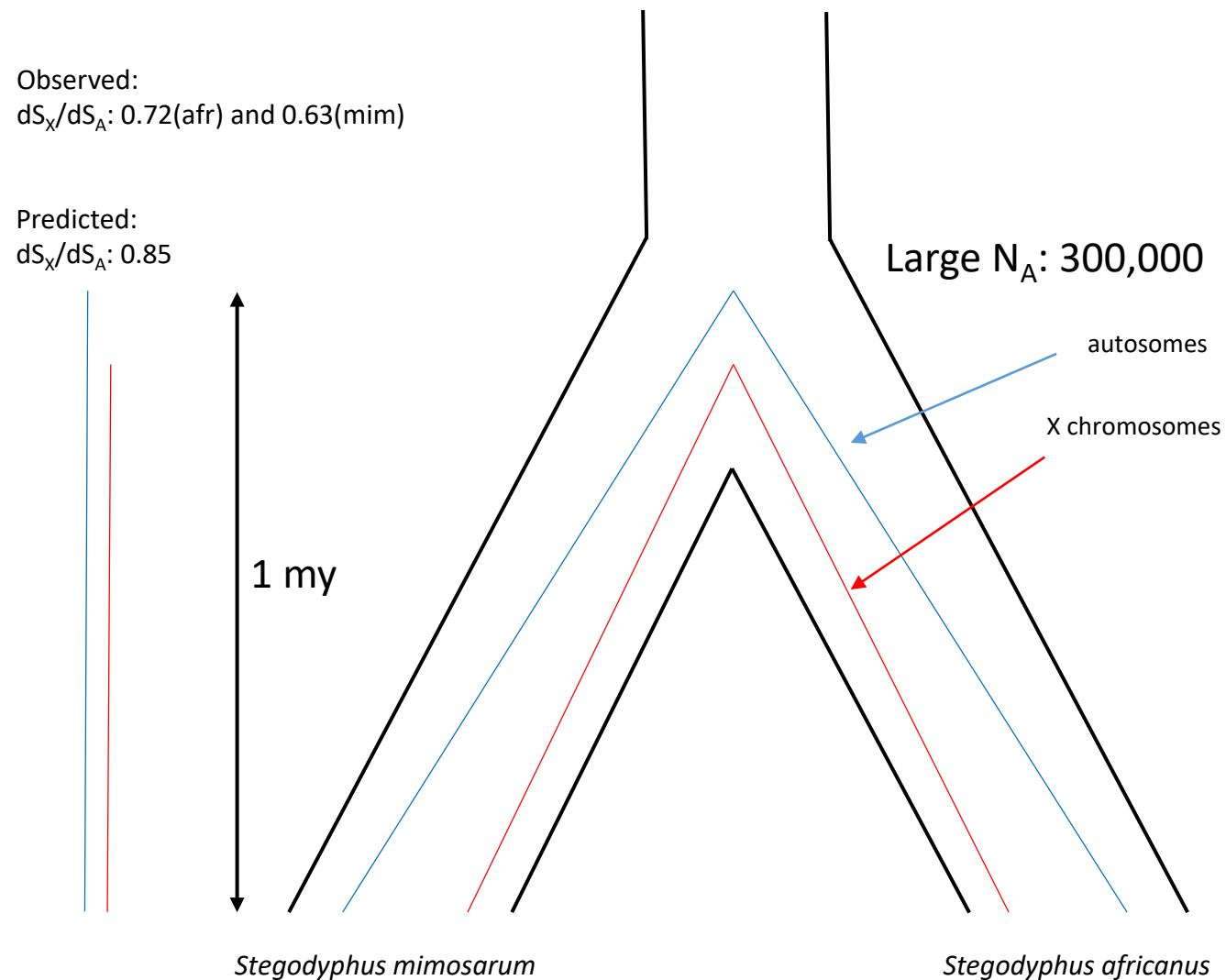

Supplement: Supplementary_Material_msz074 [file supplementary_material_msz074.zip › supplementary fig. 5.pdf]

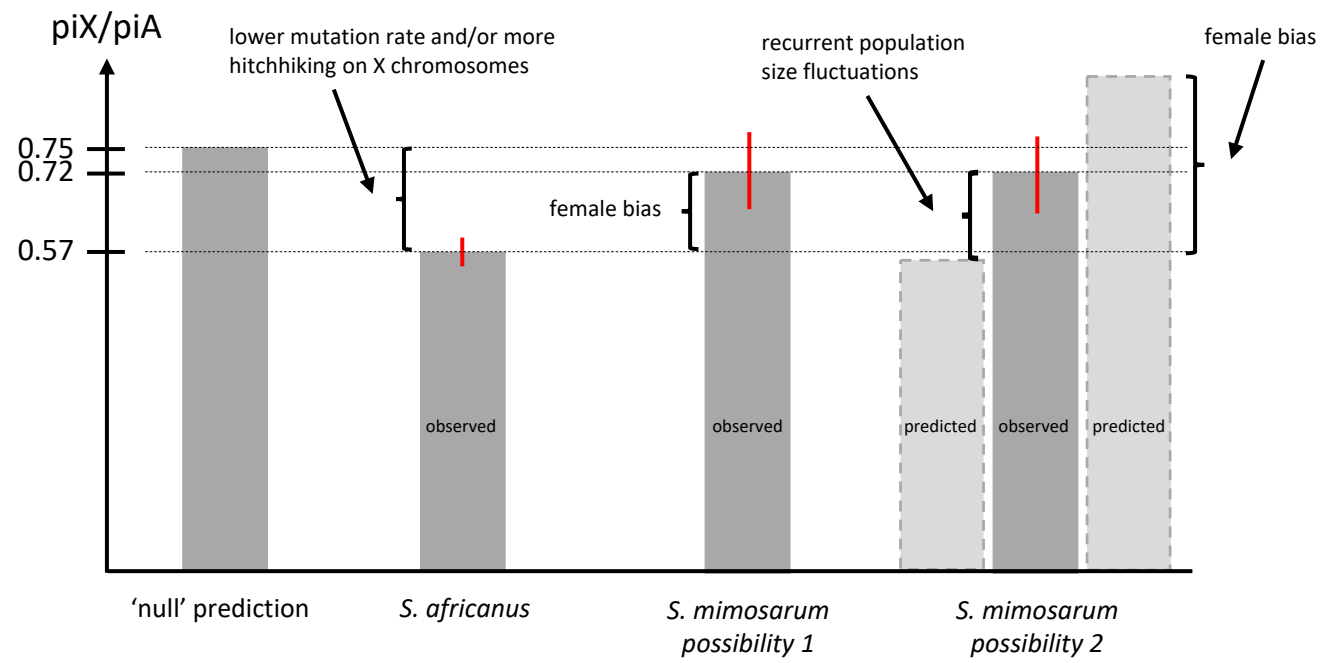

Supplement: Supplementary_Material_msz074 [file supplementary_material_msz074.zip › supplementary fig. 6.pdf]

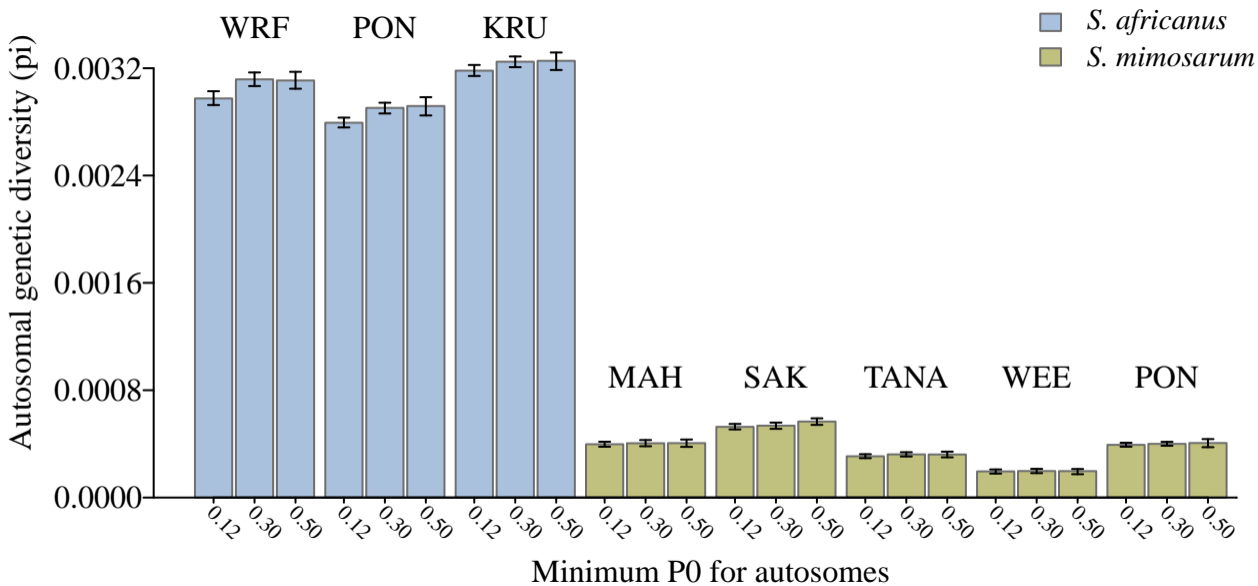

Supplement: Supplementary_Material_msz074 [file supplementary_material_msz074.zip › supplementary fig. 7.pdf]

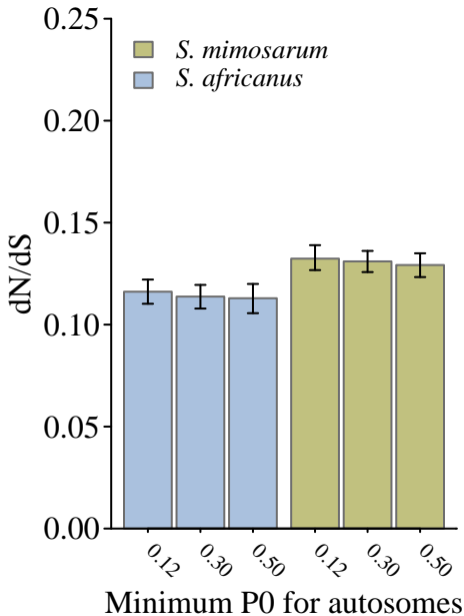

Supplement: Supplementary_Material_msz074 [file supplementary_material_msz074.zip › supplementary fig. 8.pdf]
